# Unraveling the origin of the vertebrate kidney: Emergence from a somitic compartment

**DOI:** 10.1126/sciadv.adr2618

**Published:** 2026-06-26

**Authors:** Pascal Schmidt, Eva Leman, Ketty Hakim-Mishnaevski, João E. Carvalho, Matteo Bozzo, Ronan Lagadec, Haneen Kayyal, Nadeen Dai, Boris Shklyar, Ronit Yelin, Sylvie Mazan, Michael Schubert, Ram Reshef

**Affiliations:** ^1^ Department of Evolutionary and Environmental Biology, Faculty of Natural Sciences, University of Haifa, Haifa, Israel.; ^2^ Laboratoire de Biologie du Développement de Villefranche-sur-Mer, Institut de la Mer de Villefranche, CNRS, Sorbonne Université, Villefranche-sur-Mer, France.; ^3^ Dipartimento di Scienze della Terra dell’Ambiente e della Vita, Università degli Studi di Genova, Genoa, Italy.; ^4^ Biologie Intégrative des Organismes Marins, Observatoire Océanologique de Banyuls-sur-Mer, CNRS, Sorbonne Université, Banyuls-sur-Mer, France.; ^5^ Bioimaging Unit, Faculty of Natural Sciences, University of Haifa, Haifa, Israel.; ^6^ Department of Genetics and Developmental Biology, Rappaport Faculty of Medicine, Technion–Israel Institute of Technology, Haifa, Israel.

## Abstract

Somite compartmentalization and kidney development are considered as two distinct developmental processes. It is widely acknowledged that in amniotes as well as in frogs and bony fish, the pronephros and therefore the adult kidney develop from the intermediate mesoderm. Our study sheds light on the evolution of pronephros formation in vertebrates, revealing its ancient origin from a specific, segmented, somitic subterritory, the nephrotome, which buds off during early development to create the nephric duct. In nonvertebrate chordates, mesoderm markers are expressed in common progenitor cells of dermomyotome and nephrotome, whereas in cyclostomes and elasmobranchs, they segregate into distinctive domains within the somite. Two main discoveries are thus described here: (i) The intermediate mesoderm originated developmentally and evolutionarily from a specific domain within somitic tissues, and (ii) the pronephric progenitor cell domain is redefined as the fifth somitic compartment, the nephrotome, alongside the dermatome, myotome, syndetome, and sclerotome. Our results support the hypothesis that this fifth somitic domain, the nephrotome, is the evolutionary predecessor of the intermediate mesoderm, giving rise to the pronephros and hence to the adult kidney.

## Teaser

The pronephros evolves from a somitic compartment in early vertebrates.

## INTRODUCTION

Mesoderm specification, somitogenesis, and somite compartmentalization are fundamental processes crucial for establishing the body plan of vertebrates and for orchestrating the development of key mesodermal structures, such as the muscles, bones, dermis, kidneys, gonads, heart, and appendages. Within the segmentally arranged somites, progenitor cells derived from distinct domains contribute to the formation of the dermis of the back, all striated muscles, axial bones, and tendons (*1*–*3*). The dermomyotome thus serves as the source of progenitors for the dermatome and myotome, which subsequently differentiate into the dermis and striated muscles, respectively. The sclerotome contributes to the formation of axial bones and initiates the syndetome, the fourth domain, which is composed of tendon progenitor cells (*3*, *4*). Each of these somitic domains is characterized by a specific set of transcription factors that control the early molecular events of tissue specification and differentiation (*5*–*8*). The intermediate mesoderm, a strip of tissue located lateral (in amniotes) or ventral (in amphibians and bony fish) to the paraxial mesoderm, gives rise to the excretory system and the gonads (*9*–*11*). In amniotes, the intermediate mesoderm originates from migratory cells located at a specific position along the anterior-posterior axis of the primitive streak, overlapping or posterior to the source of the paraxial mesoderm (*12*–*14*). This tissue, formerly referred to as the “intermediate cell mass,” subsequently differentiates from anterior to posterior into three distinct kidney domains: the pronephros, the mesonephros, and the metanephros (*11*, *15*, *16*). Given this anterior to posterior sequence of kidney development, different stages of nephrogenesis can thus be observed within a single embryo.

The evolutionary origin of this iterative developmental process has fascinated zoologists since the 19th century. The cephalochordate amphioxus, for example, lacks a clearly differentiated intermediate mesoderm, with the first left somite directly giving rise to the amphioxus excretory unit, Hatschek’s nephridium (*17*–*19*). On the basis of its overall morphology (*18*) and the conserved developmental expression of orthologs of two early markers of the vertebrate pronephros, *Pax2/5/8* (*20*) and *Lim1/5* (*21*), Hatschek’s nephridium has been proposed to be homologous to the vertebrate pronephros [reviewed in (*22*)]. However, there are substantial anatomical differences between Hatschek’s nephridium and the vertebrate pronephros: While the former is unpaired and characterized by a single tubule associated with clusters of filtration cells, the latter is paired and consists of a nephric duct and a set of tubules each connected to a glomerulus that serves as the main filtrating structure (*17*–*19*, *23*, *24*).

Although the paired and segmented nature of the vertebrate pronephros has already been described in the late 19th century (*25*–*27*), its mesodermal source has been a subject of debate. Early studies proposed a somitic origin for the pronephros in cyclostomes and elasmobranchs (i.e., jawless and cartilaginous fish, respectively), claiming the existence of a distinctive somitic compartment, the nephrotome, alongside the sclerotome and the myotome (*28*–*30*). While a similar organization has been proposed for amphibians (*31*), most of the reports asserted that the lateral plate mesoderm was the source of the pronephros (*23*, *26*, *27*, *32*–*36*). Subsequent studies building on these reports then defined the intermediate mesoderm as the developmental origin of the vertebrate nephric system, and anatomical evidence supporting the alternative hypothesis of a somitic origin for the kidney, in either a developmental or an evolutionary perspective, has been largely neglected.

In this study, we present molecular evidence for the emergence of the pronephros from the somites in representatives of two vertebrate clades occupying key positions in chordate phylogeny: the small-spotted catshark (*Scyliorhinus canicula*), a chondrichthyan (gnathostome vertebrate), and the European river lamprey (*Lampetra fluviatilis*), a cyclostome (jawless vertebrate). In the catshark, early expression of the pronephric kidney markers *Pax2* and *Lim1* was found within the somite in a compartment that is distinct from other somitic domains, such as the dermomyotome and the sclerotome, reminiscent of Rückert’s nephrotome (*28*). In addition, early expression of *Pax2* in the lamprey was observed within two somitic compartments, the sclerotome and a distinct ventro-lateral domain. Furthermore, we documented in both catshark and lamprey that the anterior nephrotomes bud off from the somites and merge to form the nephric duct, which extends posteriorly and can be interpreted as intermediate mesoderm in these two species. Pharmacological treatments revealed that, in contrast to amniotes, Hedgehog signaling plays a crucial role in pronephros development in both catshark and lamprey, while development of Hatschek’s nephridium in amphioxus is independent of this signaling pathway. Together, comparisons of our results from catshark and lamprey with those obtained in amphioxus allowed us to propose that a conserved anlage of the pronephros was already present in the somite of the last common ancestor of all chordates.

## RESULTS

### Somitic origin of the catshark pronephros

**Fig. 1.**
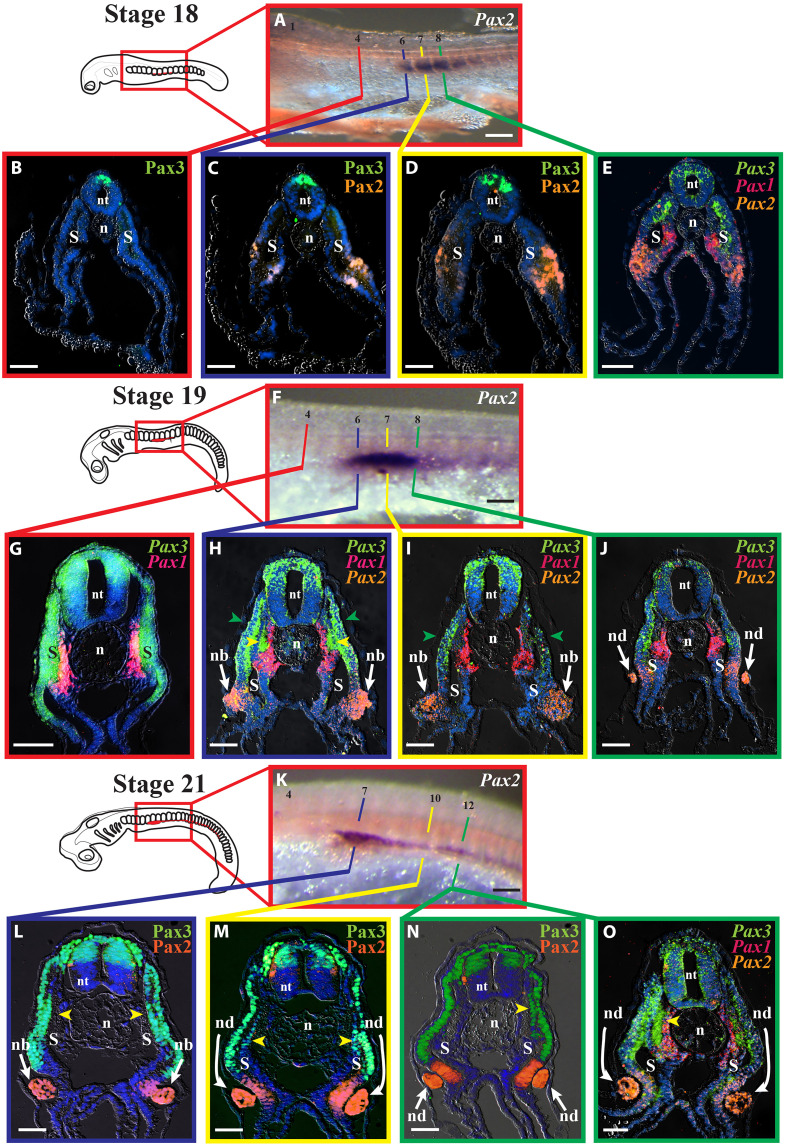
Somitic origin of the catshark pronephros. (**A**, **F**, and **K**) Enlargements of catshark (*S. canicula*) embryos in whole mounts at developmental stages 18 (*n* = 12), 19 (*n* = 25), and 21 (*n* = 18), respectively, showing colorimetric *Pax2* expression and serving as reference for the anterior-posterior position of the cross sections shown below, which were analyzed by different fluorescent staining methods. Gene names shown in italics indicate mRNA expression (and thus results of in situ hybridization assays), and gene names in regular typography indicate protein signals (and thus results of immunohistochemistry assays). Note that, with an anterior boundary at the level of mid-somite 6, different stages of pronephric development can be observed within a given embryo, depending on the level along the anterior-posterior body axis (see also figs. S2 to S4). (**B** to **E**) Cross sections of stage 18 catshark embryos at the level of the somite indicated in (A) (*n* = 15). (**G** to **J**) Cross sections of stage 19 catshark embryos at the level of the somite indicated in (F) (*n* = 24). (**L** to **O**) Cross sections of stage 21 catshark embryos at the level of the somite indicated in (K) (*n* = 20). Green arrowheads point to *Pax3* expression in the dermomyotome. Yellow arrowheads point to Pax3 expression in the myotome. In all sections, nuclear label with 4′,6-diamidino-2-phenylindole (DAPI) is shown in blue. n, notochord; nb, nephric bud; nd, nephric duct; nt, neural tube; S, somite. Scale bars, 100 μm [(A), (F), and (K)] and 50 μm [(B) to (E), (G) to (J), and (L) to (O)].

Pronephros development in the catshark *S. canicula* was initiated at late stage 17/early stage 18, based on the expression of the two early pronephric marker genes *Pax2* and *Lim1*, with an anterior expression boundary at the level of mid-somite 6, exactly as in amniotes (Fig. 1A and figs. S1 and S2) (*37*). Unexpectedly, the expression of both genes appeared segmented, suggesting localization within somites. To corroborate this hypothesis, we used immunohistochemistry, in situ hybridization chain reaction (HCR), and standard in situ hybridization and immunohistochemistry together with light sheet microscopy to define the localization of the pronephric territory relative to other mesodermal compartments. Cross sections through a stage 18 catshark embryo revealed Pax2-positive cells in ventral domains of the epithelial somite, with an anterior limit at somite 6 (Fig. 1, B to E, and fig. S2). Cross sections through somites 6 to 9 highlighted Pax2 immunoreactivity in ventro-lateral and ventro-medial domains of the somite (Fig. 1, C and D, and fig. S2, D to G), with the ventro-lateral expression domain being particularly conspicuous in somites 7 and 8 (Fig. 1, D and E, and fig. S2, E and F). At this stage, colabeling analyses identified Pax3 immunoreactivity and *Pax3* mRNA expression in the roof plate of the catshark neural tube at all axial levels analyzed, as expected (Fig. 1, B to E). In the somite, we detected expression of the *Pax3* gene at the level of somite 8, in a dorso-lateral domain (Fig. 1E), corresponding to the dermomyotome (*38*). Somitic expression of *Pax2* was neither overlapping that of *Pax1*, a marker of the sclerotome (*39*, *40*), which is limited to a medial domain (Fig. 1E), nor that of *Pax3* dorso-laterally (Fig. 1E). While the expression of all three genes was thus detectable in discrete domains of the catshark somite at stage 18, they each defined mutually exclusive somitic territories (Fig. 1E).

Similar to our observations at stage 18, multicolor in situ HCR analyses of cross sections at stage 19 revealed three distinct somitic domains comprising a *Pax2*-positive nephrotome compartment, in addition to the *Pax3*-positive dermomyotome and the *Pax1*-positive sclerotome, with *Pax3*, *Pax1*, and *Pax2* domains excluding each other (Fig. 1, F to J). At this stage, the nephric duct became morphologically distinct as an outgrowth of the ventro-lateral *Pax2*-positive somitic mesoderm (figs. S1 and S3). At the level of somites 6 and 7, pronephric development was thus characterized by the budding of *Pax2*-positive cells from the ventro-lateral epithelial somite. During the budding process, the lateral wall of the epithelial somite was disrupted, as evidenced both morphologically and molecularly (fig. S3J) (see also below). Nephric duct formation was more advanced at the level of somite 8, with a full segregation of the duct from adjacent somitic territories. At this level, *Pax2* expression was not only prominent in the newly formed nephric duct but also persisted in the ventro-lateral somite compartment (Fig. 1, G to J, and figs. S1A and S3). We found the forming nephric duct to extend posteriorly as development proceeded (Fig. 1K and figs. S1 and S4).

During subsequent development, while a nephric bud was still detectable at the level of somites 7 and 8 (Fig. 1L and fig. S4, E and F), the nephric duct was clearly visible at the level of somites 10 to 12, with Pax2 immunoreactivity persisting in the nephric bud and duct as well as in ventro-lateral cells of the somite (Fig. 1, M to O, and fig. S4). Frontal sections of *Pax2*-stained embryos at stages 20 to 24 confirmed this observation, clearly highlighting expression of the gene in the nephric duct, adjacent to lateral somitic segmented territories (fig. S1, B to E). We further identified connections of an unknown nature between the nephric duct and the somites (fig. S1, E and F, red arrows), a phenomenon that has previously been described in another shark species (*15*). Colabeling for Pax2 and Pax3 by immunohistochemistry corroborated our results obtained at earlier developmental stages, demonstrating a complete absence of Pax2 and Pax3 coexpression in the same cells (Fig. 1, L to N). The in situ HCR experiments to assess expression of *Pax3*, *Pax1*, and *Pax2* in stage 21 catshark embryos at the level of somite 12 yielded identical results (Fig. 1O). Together, these results support the hypothesis that, in the catshark *S. canicula*, the pronephros emerges from a specific, ventro-lateral domain within the somitic mesoderm, thus defining an independent somitic compartment: the nephrotome.

### The lamprey pronephros develops from somitic mesoderm

**Fig. 2.**
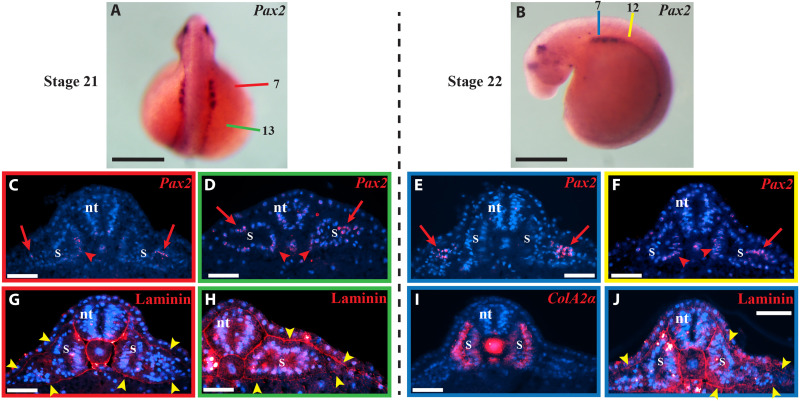
Lamprey pronephros develops from the paraxial mesoderm. (**A** and **B**) *Pax2* expression in lamprey (*L. fluviatilis*) whole-mount embryos at stages 21 and 22, respectively, as revealed by colorimetric in situ hybridization. (**C** to **J**) Cross sections at levels corresponding to colored lines in (A) (*n* = 22) and (B) (*n* = 28), with colors of the frames in (C) to (J) indicating the location of the respective cross section, at levels of somite 7 (C and G) or 13 (D and H) for stage 21 and at levels of somites 7 (E, I, and J) and 12 (F) for stage 22. Note that somite formation proceeds from anterior to posterior and that posterior somites are thus developmentally younger than anterior ones. (C to F) Expression of *Pax2* established by in situ HCR (*n* = 16), (G, H, and J) Laminin immunohistochemistry (*n* = 9), and (I) expression of *ColA2*α assayed by in situ HCR (*n* = 6). Red arrows indicate expression of *Pax2* in the lateral nephrotome, and red arrowheads indicate the expression of *Pax2* in the ventro-medial sclerotome. Yellow arrowheads indicate the borders of the paraxial mesoderm as defined by Laminin staining. In all sections, nuclear label with DAPI is shown in blue. nt, neural tube; S, somite. Scale bars, 200 μm [(A) and (B)] and 30 μm [(C) to (J)].

In the lamprey, both *Pax2* and *Lim1* are expressed in the developing pronephros, but *Pax2* expression is detectable significantly earlier (at stage 21) than that of *Lim1* (at stage 24) (*37*). We thus analyzed early nephric development in the lamprey (*L. fluviatilis*) using *Pax2* as the earliest available pronephros marker. Since in lamprey embryos, especially at the developmental stages pertinent for this study, autofluorescence is significant in both green and orange wavelengths, only the far-red spectrum is leaving to be exploited by fluorescent imaging. We could thus not multiplex our expression analyses and therefore used embryos at the exact same developmental stage to analyze each gene marker individually. As in the catshark, the earliest expression of *Pax2* in the trunk, observed at stages 21 and 22, was segmented, with an anterior limit precisely corresponding to the level of somite 6 (Fig. 2, A and B, and fig. S5, A and F) (*30*, *37*). Cross sections demonstrated that *Pax2*-positive cells were located within the somites, on each side of the well-individualized neural tube and notochord (Fig. 2, C to F, and fig. S5, B to E) and defined by two microstructural parameters of developing somites: (i) the cellular organization, revealed by 4′,6-diamidino-2-phenylindole (DAPI) staining, and (ii) the extracellular matrix, visualized by Laminin (Fig. 2, G to J) (*41*). Mapping *Pax2* expression relative to DAPI staining and the Laminin signal showed that *Pax2* expression was restricted to two separate domains within the somite, respectively located laterally and ventro-medially (Fig. 2, C to J). While *Pax2* was conspicuously expressed in both the lateral and the ventro-medial domains of posterior (and thus developmentally younger) somites, its expression was weaker in ventro-medial territories of anterior (and thus developmentally older) somites (Fig. 2, C to F).

Correlation of *Pax2* expression with that of the sclerotome marker *LecD* (*42*) suggested that the ventro-medial expression of *Pax2* was within the sclerotomal domain (fig. S5J). Furthermore, expression of the sclerotomal marker *Sox9* (*43*) overlapped that of *Pax2* in the lateral domain at stage 22 (fig. S5G). We also found that the domain of *Col2*α*1a* expression, which marks the dermomyotome and sclerotome (*43*), was distinct from that of *Pax2* at the level of somite 7 at stage 22 (Fig. 2, E and I). These results are consistent with the notion that the lateral domain of *Pax2* corresponds to a separate compartment within the somite (Fig. 2, E, I, and J). This separation was much less evident at stage 21 as well as in more posterior somites of stage 22 (early stage somites) (Fig. 2, C, D, and F to H). We further corroborated these results by colabeling analyses of *Pax2* with the dermomyotome marker *Pax3/7* (*44*), combining in situ HCR for *Pax2* with colorimetric in situ hybridization for *Pax3/7*, which revealed a clear spatial separation of *Pax2*- and *Pax3/7*-positive cells within the somites at stages 21 and 22 (fig. S5, H and I). Together, this suggests that the lamprey pronephros originates from a specific domain within the somite, the nephrotome, and starts separating from this tissue at stage 22, in a process that is initiated at the level of somite 6 and subsequently progresses posteriorly as development proceeds.

### Hatschek’s nephridium in amphioxus is derived from the first left somite

**Fig. 3.**
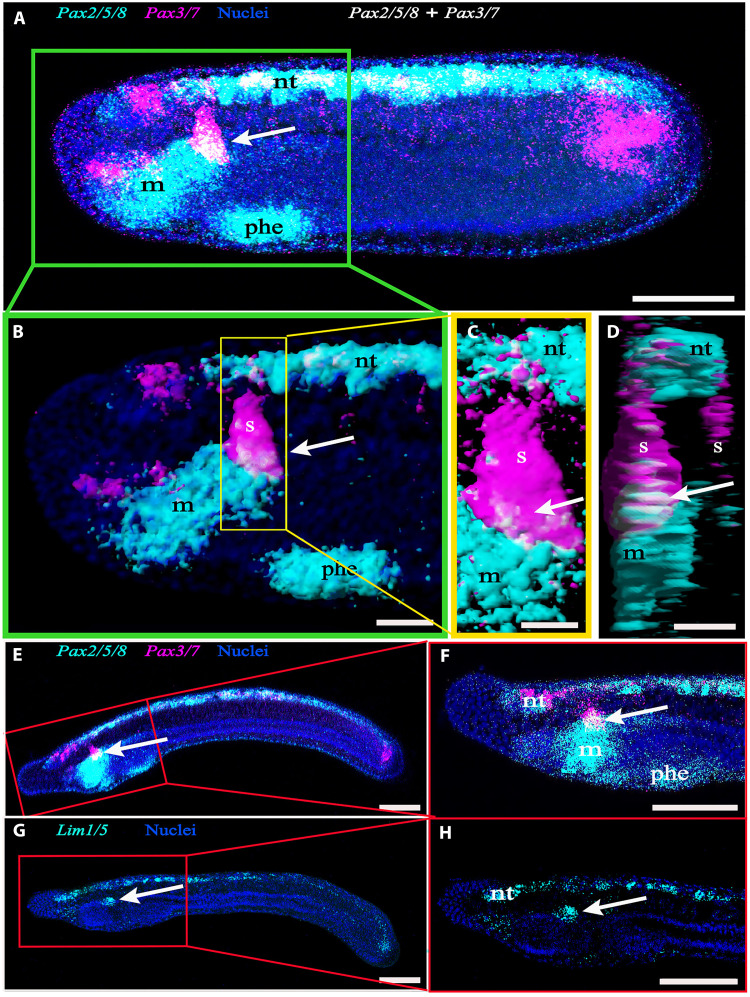
Amphioxus Hatschek’s nephridium develops from the first left somite. (**A** to **D**) Expression of *Pax2/5/8* and *Pax3/7* in whole-mount N5-stage embryos of the amphioxus *B. lanceolatum*, as revealed by in situ HCR. Maximum intensity projection in the left side view with anterior to the left (*n* = 6). (B) 3D reconstruction of the region in the boxed area in (A), showing the anterior region of the embryo on the left side. (C) Magnification of the boxed area in (B), highlighting the region of the first somite. (D) Rotation of the reconstruction of the boxed area in (C), displaying a transverse section at the level of the first somite. Arrows highlight the location of Hatschek’s nephridium. Coexpression of *Pax2/5/8* and *Pax3/7* is shown in white. (**E** and **F**) Expression of *Pax2/5/8* and *Pax3/7* in whole-mount L0-stage larvae of *B. lanceolatum*, as revealed by in situ HCR. Maximum intensity projection in the left side view with anterior to the left (*n* = 10). (F) Magnification of the boxed area in (E), showing the anterior region of the larva. Coexpression of *Pax2/5/8* and *Pax3/7* is shown in white. (**G** and **H**) Expression of *Lim1/5* in whole-mount L0-stage larvae of *B. lanceolatum* at L0 stage, as revealed by in situ HCR. Maximum intensity projection in the left side view with anterior to the left (*n* = 10). (H) Magnification of the boxed area in (G), highlighting the anterior region of the larva. Nuclear staining by Hoechst is shown in dark blue. m, location of the future mouth; nt, neural tube; phe, pharyngeal epithelium; S, somite. Scale bars, 50 μm (A), 20 μm (B), 10 μm [(C) and (D)], and 50 μm [(E) to (H)].

In the European amphioxus *Branchiostoma lanceolatum*, the anlage of Hatschek‘s nephridium in the first left somite (*17*) expresses both *Pax2/5/8* (*20*) and *Lim1/5* (*21*), which are the amphioxus orthologs of the vertebrate pronephric markers *Pax2* and *Lim1*, respectively. At the N5 neurula stage (*45*), *Pax2/5/8*-positive cells were first detected in the ventro-lateral compartment of the first left somite (Fig. 3, A to C) but not in the first right somite (Fig. 3D and fig. S6) (*20*). To localize the *Pax2/5/8* signal within the somite, we colabeled N5-stage embryos with *Pax2/5/8* and *Pax3/7*, a gene expressed centro-laterally in the dermomyotomal compartment of amphioxus somites (*46*) as well as in the first left somite at late neurula and early larval stages (*47*). As reported in (*47*), we found broad expression of *Pax3/7* in the first left somite of the N5-stage embryo (Fig. 3, A to D). Unexpectedly, the ventral-most portion of the *Pax3/7* domain in the first left somite overlapped the expression of *Pax2/5/8* (Fig. 3, A to D). While *Pax3/7* expression was largely down-regulated in other somites, coexpression of *Pax3/7* and *Pax2/5/8* in the first left somite persisted at the L0 stage (Fig. 3, E and F), when *Lim1* was also detectable in Hatschek’s nephridium (Fig. 3, G and H). The persistent expression of *Pax3/7* in the first left somite suggests that the developmental program for patterning this somite is different from that of other somites. In addition, this result indicates that the first left somite of amphioxus is dermomyotomal. Consistent with this notion, *ColA*, which marks nonmyotomal somite derivatives (*46*, *48*), is specifically absent from the first left somite (fig. S7). The fact that *Pax2/5/8*, a pronephric marker, and *Pax3/7*, a dermomyotome marker, are coexpressed in cells of the first left somite in amphioxus is reminiscent of our results in the lamprey, where *Pax2* expression in somitic mesoderm might not be restricted solely to the nephrotome.

### Establishment of the nephric duct in the catshark

**Fig. 4.**
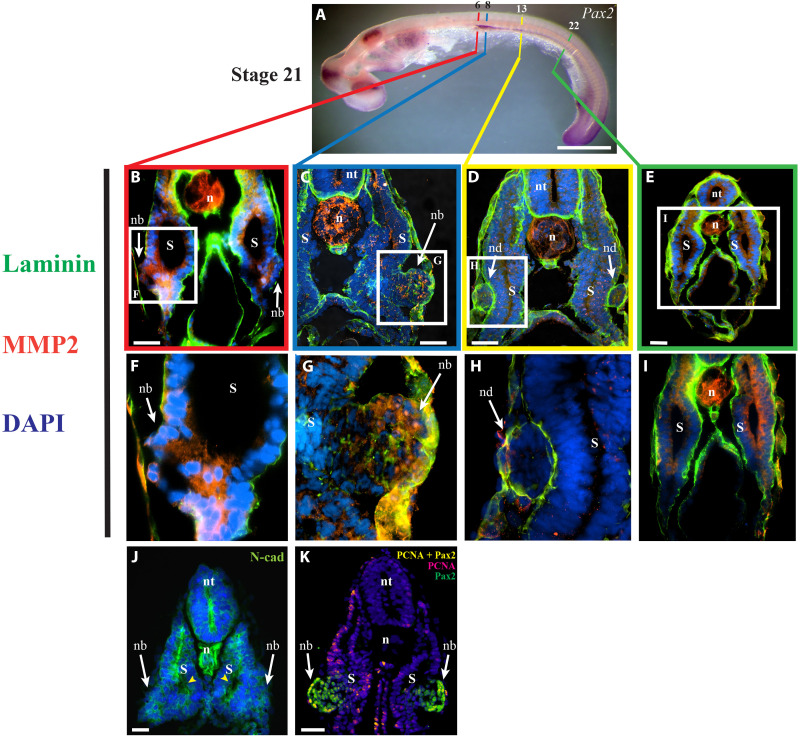
The establishment of the nephric duct in the catshark. (**A**) Colorimetric whole-mount in situ hybridization of a catshark (*S. canicula*) embryo at stage 21 showing *Pax2* expression (*n* = 12) and serving as reference for the anterior-posterior position of the sections shown below. Note that, depending on the level along the anterior-posterior body axis, different stages of pronephric development can be observed in the stage 21 catshark embryo. Colored lines and boxes denote cross sections in (B) to (E), respectively, at levels of somites 6, 8, 13, and 22. (**B** to **E**) Cross sections showing the distribution of Laminin (green) and MMP2 (red) obtained by immunohistochemistry (*n* = 4). (**F** to **I**) Magnifications of the boxed areas in, respectively, (B) to (E). (**J**) N-cadherin (N-cad) immunohistochemistry (green) (*n* = 3). (**K**) PCNA (pink) and Pax2 (green) immunohistochemistry showing cell proliferation in the distal region of the bud (yellow) (*n* = 5). In all sections, nuclear label with DAPI is shown in blue. n, notochord; nb, nephric bud; nd, nephric duct; nt, neural tube; S, somite. Scale bars, 500 μm (A) and 50 μm [(B) to (E), (J), and (K)].

Following the observation of nephric duct budding from a ventro-lateral somitic domain in the catshark, we hypothesized that this cytological process involves epithelial to mesenchymal transition followed by mesenchymal to epithelial transition. To assess this hypothesis, we took advantage of the fact that different stages of pronephric development are observable along the anterior-posterior body axis of a given embryo. Therefore, in a catshark embryo at stage 21, budding is taking place at the level of somite 6, creation of the duct and a completely closed duct are observable at respectively more posterior levels, while the most posterior somites completely lack pronephric structures. Using immunohistochemistry targeting known markers of epithelial cellular phenotypes, such as the metalloprotease matrix metalloproteinase 2 (MMP2), N-cadherin, and Laminin, we analyzed nephric duct formation in catshark embryos at stage 21 (Fig. 4A). We found that the epithelial somite was surrounded by Laminin and that MMP2 and N-cadherin were restricted to the apical poles of epithelial somitic cells (Fig. 4, B to J). However, at the level of somite 6 (i.e., at the site of nephric duct budding), Laminin and N-cadherin distribution was disrupted (Fig. 4, B, F, and J), and MMP2 was accumulated at the budding site, with its signal persisting at the apical poles of epithelial somitic cells (Fig. 4, B and F). At the level of somite 8, the budding process was more advanced. Laminin expression was inconspicuous around the bud, and MMP2 was detectable throughout the bud but with no significant connection to the MMP2 signal in the epithelial cells of the somite (Fig. 4, C and G). Double immunostaining for the cell proliferation marker proliferating cell nuclear antigen (PCNA) and Pax2 revealed that Pax2-positive cells in the distal region of the bud also expressed PCNA, suggesting that cell proliferation is taking place in the distal pronephric bud (Fig. 4K). At the level of somite 13, the nephric duct was fully formed, and its epithelial structure was surrounded by Laminin, with weak expression of MMP2 in its center (Fig. 4, D and H). This fully established nephric duct is the result of the fusion and posterior migration of the buds related to somites 6 to 9, as shown in figs. S1 (A to F) and S3J. At even more posterior levels, such as at the level of somite 22, there were no pronephric structures, and the epithelial somites were surrounded by Laminin, with MMP2 being detectable at the apical pole of the somitic cells (Fig. 4, E and I). Together, these results suggest that, in the catshark embryo, the nephric bud initially emerges from the epithelial somite by epithelial to mesenchymal transition, involving cell proliferation at the distal budding region and that the epithelial structure of the nephric duct is subsequently established by mesenchymal to epithelial transition.

### Hedgehog signaling is necessary for the development of the pronephros in catshark and lamprey, but not for the formation of Hatschek’s nephridium in amphioxus

**Fig. 5.**
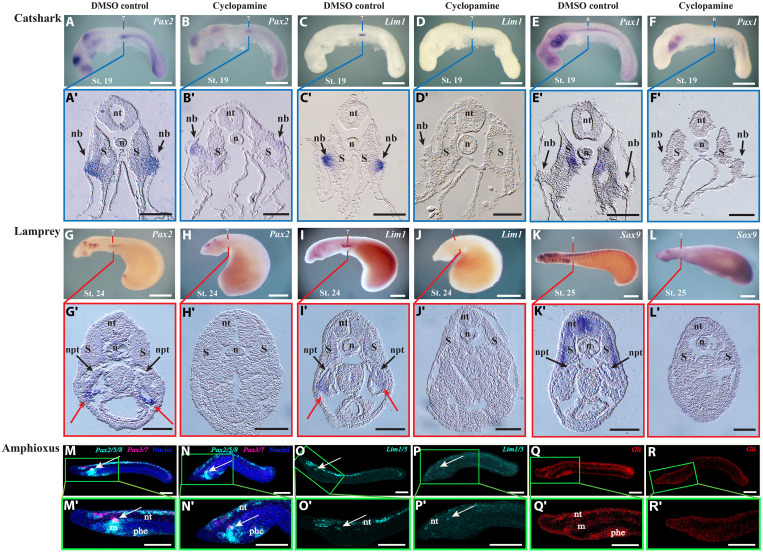
Hedgehog signaling is necessary for the development of the catshark and lamprey pronephros. (**A** to **F**) Colorimetric in situ hybridization of whole-mount catshark embryos (*S. canicula*) at stage 19, following pharmacological treatments, in the left side view and with anterior to the left. (A, C, and E) Treatments with the control solvent DMSO. (B, D, and F) Treatments with cyclopamine, a Hedgehog signaling inhibitor. (A and B) Expression of *Pax2* (DMSO control *n* = 4/4, cyclopamine *n* = 9/10). (C and D) Expression of *Lim1* (DMSO control *n* = 3/3, cyclopamine *n* = 5/5). (E and F) Expression of *Pax1* (DMSO control *n* = 3/3, cyclopamine *n* = 4/4). (A′ to F′) Cross sections through the pronephros domain of catshark embryos at the somite levels indicated, respectively, in (A) to (F), also highlighted by colored lines and boxes. (**G** to **L**) Colorimetric in situ hybridization of whole-mount lamprey embryos (*L. fluviatilis*) at stage 24 after pharmacological treatments, in the left side view and with anterior to the left. (G, I, and K) Treatments with the control solvent DMSO. (H, J, and L) Treatments with cyclopamine, a Hedgehog signaling inhibitor. (G and H) Expression of *Pax2* (DMSO control *n* = 15/15, cyclopamine *n* = 10/10). (I and J) Expression *Lim1* (DMSO control *n* = 11/11, cyclopamine *n* = 12/12). (K and L) Expression of *Sox9* (DMSO control *n* = 9/9, cyclopamine *n* = 8/8). (G′ to L′) Cross sections through the pronephros domain of lamprey embryos at the somite levels indicated, respectively, in (G) to (L), also highlighted by colored lines and boxes. (**M** to **R**) Whole-mount in situ HCR analysis of amphioxus embryos (*Branchostoma lanceolatum*) at the L0 stage after pharmacological treatments, in the left side view and with anterior to the left. (M, O, and Q) Treatments with the control solvent DMSO. (N, P, and R) Treatments with cyclopamine, a Hedgehog signaling inhibitor. (M and N) Expression of *Pax2/5/8* and *Pax3/7* (DMSO control *n* = 8/8, cyclopamine *n* = 10/10). Arrows highlight Hatschek’s nephridium. *Pax2/5/8* and *Pax3/7* coexpression is shown in white. (O and P) Expression of *Lim1/5* (DMSO control *n* = 9/9, cyclopamine *n* = 10/10). Arrows highlight Hatschek’s nephridium. (Q and R) Expression of *Gli* (DMSO control *n* = 9/10, cyclopamine *n* = 10/10). (M′ to R′) Scans to magnify the anterior regions of the larvae respectively shown in (M) to (R), illustrated by colored lines and boxes. In (M′) to (P′), arrows highlight Hatschek’s nephridium. Nuclear staining by Hoechst in (M), (N), (M′), and (N′) is shown in dark blue. m, location of the future mouth; n, notochord; nb, nephric bud; npt, nephric tubule; nt, neural tube; phe, pharyngeal epithelium; S, somite. Scale bars, 400 μm [(A) to (F)], 90 μm [(Aʹ) and (Fʹ)], 200 μm [(G) to (L)]. 60 μm [(Gʹ) to (Lʹ)], 50 μm [(M) to (R)], and 50 μm [(Mʹ) and (Rʹ)].

The expression of *Pax2* in the ventro-lateral somitic mesoderm in catsharks and lampreys and the transient expression of *Pax2* in a ventro-medial domain of the somite in lampreys raised the possibility that expression of this gene in both species is controlled by Hedgehog signaling, which is known to control determination of the paraxial mesoderm in other vertebrates (*49*, *50*). To test this hypothesis, we used pharmacological treatments with cyclopamine (details in Materials and Methods) to inhibit Hedgehog signaling during catshark and lamprey development and subsequently assessed expression of the nephrotome markers *Pax2* and *Lim1* as well as of the sclerotome markers *Pax1* (in the catshark) and *Sox9* (in the lamprey) and of the dermomyotome marker *Pax3* (in the catshark). We defined a precise cyclopamine concentration and specific time window to affect pronephros development. Injection of 200 μM cyclopamine into the eggshell of stage 12 or 13 embryos thus resulted in severe defects in pronephros development (40 of 51), while injections into stages 14 and above resulted in no discernible pronephros phenotypes (8 of 8). Treatments affecting pronephros development also produced a shortening of the head, a deformation of the somites and neural tube as well as, in most cases, a malformed tail (Fig. 5, A to F and A′ to F′). Detailed morphological and anatomical analyses revealed significantly reduced pronephric buds, which were shifted posteriorly by two to four somites. Molecular analyses showed that the treatments induced a marked reduction of *Pax2* and a complete loss of *Lim1* expression (Fig. 5, A to D and A′ to D′). The treatments also led to a complete down-regulation of *Pax1* expression in the sclerotomal domains of the somite (Fig. 5, E, E′, F, and F′, and fig. S8), which is consistent with results from other vertebrates, where *Pax1* acts downstream of Hedgehog signaling during sclerotome development (*49*, *51*, *52*). As expected, the cyclopamine treatments thus repatterned the somites and neural tube by extending *Pax3* expression into ventral regions of these tissues. At the level of somite 8, this even included the nephric bud, where *Pax2* was completely abolished (fig. S8E). Of note, while a reduction of *Pax2* expression was observable at all assayed anterior-posterior levels of the budding region, we never detected *Pax2* in any other somitic territory (fig. S8, white arrowheads).

Treatment of lamprey embryos with cyclopamine starting at stage 12 also induced severe malformations, including a significant reduction in trunk size and abnormal pronephros development, with a complete absence of nephric tubule formation (Fig. 5, G to L and G′ to L′). Somitic expression of both *Pax2* and *Lim1* was thus completely lost in treated embryos, while *Pax2* expression in the mid-hindbrain boundary and otic vesicle was normal (Fig. 5, G to J and G′ to J′). Similar to the results obtained in catsharks, the treatments also led to a down-regulation of the expression of the sclerotome marker *Sox9* (Fig. 5, L and L′), which, in amniotes, is known to be under the control of Hedgehog signaling (*53*, *54*). Together, these results demonstrate that, as in other vertebrates, Hedgehog signaling is required for the differentiation of somitic mesoderm in both catsharks and lampreys. However, our experiments further indicate that in chondrichthyans and cyclostomes, this regulation includes a specific ventro-lateral compartment within the somite: the nephrotome.

Because of the somitic origin of Hatschek’s nephridium in cephalochordates, we also performed cyclopamine treatments at mid-gastrula stages in amphioxus. Inhibition of Hedgehog signaling led to severe malformations as well as to a general reduction of larval size. In addition, expression of *Gli*, which is known to be directly regulated by Hedgehog signaling (*55*–*57*), was strongly reduced, most notably in neural tube and pharynx (Fig. 5, M to R and M′ to R′). However, in Hatschek’s nephridium, the treatments did neither affect the coexpression of *Pax2/5/8* and *Pax3/7* (Fig. 5, M, N, M′, and N′) nor the expression of *Lim1/5* (Fig. 5, O, P, O′, and P′). These results suggest that the development of Hatschek’s nephridium from the first left amphioxus somite does not require Hedgehog signaling activity.

## DISCUSSION

**Fig. 6.**
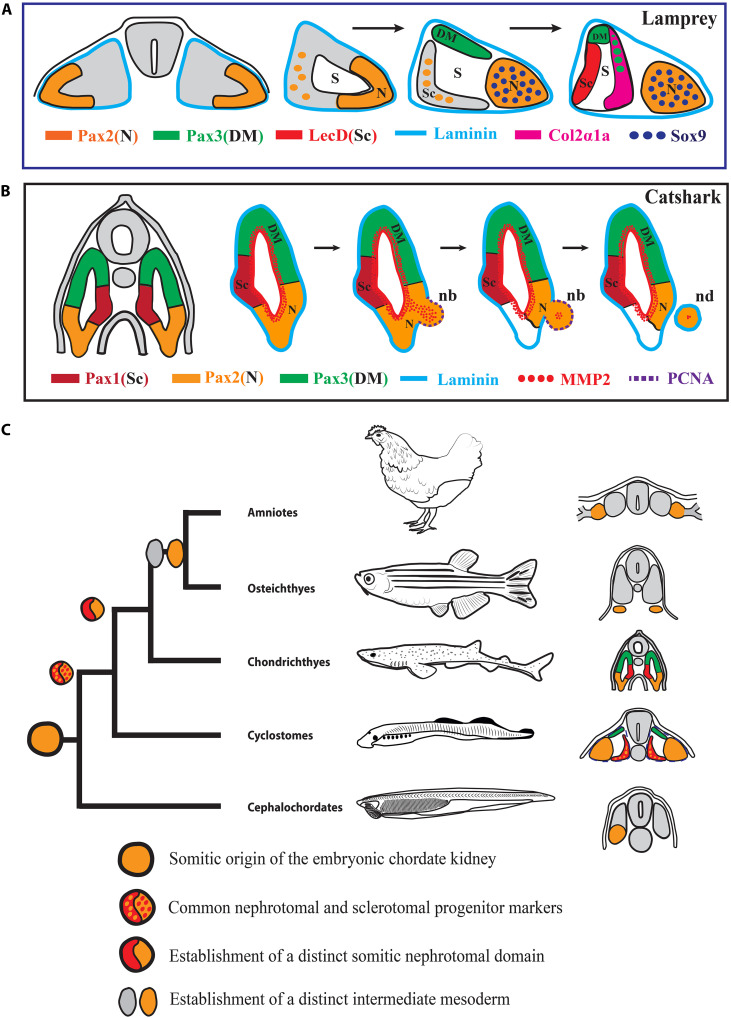
Development and evolution of the pronephros in chordates. Developmental sequences and evolutionary scenario are derived from observations in catshark, lampreys, and amphioxus. For catshark and lamprey, different developmental stages and different levels along the anterior-posterior body axis have been used to define the progression of pronephric development. As formation of the pronephros follows an anterior to posterior sequence, different stages of pronephros development can be observed within a given embryo. (**A**) Specification of the nephrotome in the lamprey, from a domain within the paraxial mesoderm. (**B**) The nephrotome in the catshark, localized within a ventro-lateral domain of the somite, and the process of nephric budding. (**C**) A phylogenetic tree of chordates presenting a scenario for the evolution of the vertebrate kidney. DM, dermomyotome; N, nephrotome; nb, nephric bud; nd, nephric duct; S, somite Sc, sclerotome.

In this study, we characterized the development of the pronephros in three species at key positions of chordate phylogeny: the small-spotted catshark (*S. canicula*), a chondrichthyan, the European river lamprey (*L. fluviatilis*), a cyclostome, and the European amphioxus (*B. lanceolatum*), a cephalochordate. In situ hybridization and immunohistochemistry assays combined with pharmacological treatments provided strong evidence for the existence, in the three species, of an early pronephric territory that forms as an integral compartment of the somite, in a ventro-lateral position. A somitic origin of the nephrotome might thus represent an ancestral chordate feature, retained in cephalochordates, cyclostomes, and chondrichthyans but lost in amniotes. The somitic nephrotome might thus represent an evolutionarily conserved entity that predates the emergence of an intermediate mesoderm (Fig. 6).

### Evolution of pronephric progenitor states in chordates

The role of the cephalochordate amphioxus in understanding the origin and evolution of the vertebrate kidney has been a subject of debate for many decades. Early comparative anatomists compared the branchial nephridia of amphioxus adults with the vertebrate pronephros (*23*, *30*, *58*). These comparisons were primarily based on the segmented nature of both structures, although the segmentation in amphioxus is branchiomeric rather than somitomeric. It was argued that the anterior somites of vertebrates were originally associated with the gill slits and therefore with the branchial nephridia. However, another kidney-like structure of amphioxus, Hatschek’s nephridium, has been proposed as a potential homolog of the vertebrate pronephros [reviewed in (*22*)]. Hatschek’s nephridium consists of an unpaired tubule and develops from the ventro-lateral portion of the first left somite, which expresses homologs of the vertebrate pronephric kidney markers *Pax2* and *Lim1* (*17*, *20*, *21*). We show here that expression of the amphioxus *Pax2* homolog overlaps with that of the amphioxus *Pax3* homolog, which marks the dermomyotomal compartment of amphioxus somites (*46*). This suggests that the ventro-lateral domain of the first left cephalochordate somite accommodates identities of at least three vertebrate-type mesodermal progenitor pools: the nephrotome, the dermatome, and the myotome. Whether this lack of a strict separation of mesodermal progenitor cell populations represents an ancestral chordate trait remains to be established. Nevertheless, the transient expression, in the lamprey somite, of both *Pax2* and *Sox9* ventro-laterally (i.e., in the nephrotome) and of *Pax2* ventro-medially (i.e., in the sclerotome) (Fig. 6A) is at least reminiscent of the situation observed in amphioxus and thus suggests the existence of a bipotential progenitor cell population in lampreys, contributing to both sclerotome and nephrotome.

Differences in the specification process of mesoderm progenitor populations might also explain the results of our pharmacological treatment experiments targeting the activity of the Hedgehog signaling pathway in catsharks and lampreys. In the catshark, treatment with cyclopamine to block Hedgehog signaling activity resulted in pronephric malformations as well as in a posteriorization of the budding process. Expression of *Lim1* in the ventro-lateral somite was lost and that of *Pax2* was attenuated. In addition, expression of the sclerotome marker *Pax1* was no longer detectable in the ventro-medial somite while that of the dermomyotome marker *Pax3* was expanded ventrally in the somite. These results suggest that, in the catshark, Hedgehog signaling plays an important role in the specification of somitic territories. In particular, the down-regulation, following cyclopamine treatments, of both sclerotomal and nephrotomal markers at the expense of dermomyotome markers is indicative of a Hedgehog-dependent regulatory mechanism that concomitantly promotes sclerotomal and nephrotomal fates. Concerning subsequent pronephros formation, since we observed pronephric budding even in the absence of *Lim1*, it seems that *Lim1* is dispensable for the budding process in the catshark and may hence only be required at later stages of pronephric development. Last, the posteriorization of the budding process we identified in cyclopamine-treated embryos might be explained by the presence of an anterior-posterior activity gradient of Hedgehog signaling in the embryo, a notion that will require further work to be corroborated.

Cyclopamine treatments of lamprey embryos resulted in a complete loss of *Pax2* and *Lim1* expression in the ventro-lateral somite, which was accompanied by an absence of pronephric structures. Given that the treatments abolished expression of sclerotome markers in both species (*Pax1* in catsharks and *Sox9* in lampreys), these results suggest that, while Hedgehog signaling controls sclerotome development in both catsharks and lampreys, as it does in other vertebrates (*49*, *50*), the Hedgehog dependence of pronephros development may slightly differ between the two species. Hedgehog signaling is thus required for the earliest stages of pronephric budding in lampreys and seems to act only later in catsharks, possibly by differential control of *Pax2* and *Lim1* expression. It might be that these differences are related to the lack of separation of mesodermal progenitor cell populations in lampreys. The evolution of this separation might have provided the basis for the differential regulation of progenitor specification by Hedgehog signaling in the catshark somite.

The regulation of the formation of Hatschek’s nephridium in amphioxus is known to be different from that of the vertebrate pronephros. For instance, the development of Hatschek’s nephridium is not dependent on retinoic acid signaling, which contrasts the situation in gnathostomes (*37*). Consistent with this notion, our results indicate that Hedgehog signaling is not required for the development of Hatschek’s nephridium. This lack of Hedgehog signaling dependence may be correlated with the absence of a sclerotome compartment in the first left amphioxus somite. Sclerotome development is known to depend on Hedgehog signaling (*49*–*54*), a condition that appears to be linked to nephrotome formation in lampreys and sharks. A connection between sclerotomal and nephrotomal cell lineages might have thus been established during early vertebrate diversification, through the gain of Hedgehog signaling dependence.

In amniotes, there is no evidence for an involvement of Hedgehog signaling in pronephros development. Instead, signaling factors emanating from the dorsal neural tube and the lateral plate mesoderm (and not from ventral midline tissues, such as the ventral neural tube or the notochord, which are known sources of Hedgehog signaling ligands) induce pronephros markers in competent intermediate mesoderm (*11*, *16*, *59*, *60*). Moreover, mice lacking the *Sonic hedgehog* (*Shh*) gene exhibit severe somitic defects, particularly in the sclerotome, significant abnormalities in the central nervous system, and malformations in the metanephric kidney as well as in several other internal organs (*61*). However, the fact that metanephric kidneys develop in *Shh* mutant mice suggests that pronephros development is not affected (*62*), indicating that Hedgehog signaling is not necessary for the formation of the pronephros in mice. Together, these findings suggest that the involvement of Hedgehog signaling in pronephros development might be an ancestral trait of vertebrates that has subsequently been lost, at least in the lineage leading to extant amniotes.

### An ancient somitic origin of the pronephros: From nephrotome to intermediate mesoderm

The vertebrate kidney is presently regarded as a derivative of the intermediate mesoderm (*11*, *14*, *16*, *62*–*64*). From a historical perspective, however, Rückert’s seminal study in 1888 examined the development of the pronephros in various shark species and initially proposed its origin from somitic tissue (*28*). Rückert thus described the development of the pronephros in sharks from a compartment located within the ventro-lateral somite, which he termed the nephrotome, to complement the already recognized myotome and sclerotome (*28*). Following Rückert’s investigations, some of his contemporaries supported his findings (*29*, *65*), while others contended that the segmented pronephros arises as a diverticulum of the unsegmented portion of the peritoneal cavity or the parietal peritoneum, which is derived from the somatopleura (*25*–*27*, *32*). The developmental origin of the pronephros from this intermediate cell mass, which lies adjacent to the somites, was subsequently adopted by the scientific community, and the intermediate cell mass was termed the intermediate mesoderm (*15*).

Our results, however, demonstrate that in the catshark, a ventro-lateral domain within the somite, with an anterior limit at the level of somite 6 (*37*), expresses the vertebrate pronephric kidney markers *Pax2* and *Lim1*. This *Pax2*- and *Lim1*-positive somitic domain is distinctly separate from the *Pax1*- and *Sox9*-expressing sclerotome and the *Pax3*-expressing dermomyotome (Fig. 6B). This finding suggests that the catshark somite is composed of five somitic compartments, dermatome, myotome, sclerotome, syndetome, and nephrotome (*40*, *66*, *67*), which is in line with Rückert’s initial observations on pronephric development in sharks (*28*). We further observed that the *Pax2*- and *Lim1*-positive somitic domains in the catshark embryo undergo lateral evaginations through a budding process and subsequently fuse to give rise to the pronephric duct (Fig. 6B), as described in the early literature (*28*, *29*).

Similar to the situation in catsharks, early studies on the mechanisms of pronephric development in lampreys and other cyclostomes controversially described its embryonic origin either from somitic tissue (*30*, *68*) or from lateral plate mesoderm (*23*, *33*–*35*). We found segmentally arranged expression of the pronephric kidney markers *Pax2* and *Lim1* along the anterior-posterior body axis of the lamprey embryo (*37*), with cross sections revealing *Pax2* expression in a ventro-lateral domain within the somite, in close proximity to the sclerotome medially and the dermomyotome dorso-laterally (Fig. 6A). However, while in the catshark *Pax1* plus *Sox9* (fig.S1, I and J), *Pax3*, and *Pax2* plus *Lim1* define distinct, mutually exclusive territories, *Pax2* in the lamprey is also expressed in sclerotomal domain and *Sox9* transiently in the nephrotome domain during early somite specification. This observation in lampreys suggests the presence of common progenitor cells for sclerotome and nephrotome that may represent an early vertebrate trait. In addition, the presence of a pronephros progenitor domain within the lamprey somite (*30*) can be likened to Rückert’s concept of the shark nephrotome.

In amphibians, specifically in the order Gymnophiona, an elongated, segmentally arranged pronephros is visible along the embryonic body, with each segment supposedly derived from somitic tissue (*15*). According to Brauer (*31*), the Gymnophiona pronephros emerges from a ventro-lateral domain of the somite, similar to the shark pronephros. Furthermore, transplantation experiments in urodeles, grafting paraxial mesoderm from a neurula stage embryo into the ventral trunk of another embryo at the same stage, revealed that the graft develops into pronephric tubuli (*69*). This suggests that the urodele somite, when isolated from its neighboring tissues, is sufficient to adapt a pronephric fate. Despite these observations, however, the existence of a nephrotomal somitic compartment, at least in certain vertebrate clades, has not gained widespread acceptance. Considering the previous reports and our current results, this lack of acceptance needs to be revisited.

### Evolution of the chordate pronephros

On the basis of the data reported here, we propose a scenario for the evolution of pronephros development in chordates (Fig. 6C). This scenario stipulates a somitic origin of the pronephros, with a nephrotome representing an ancestral chordate trait. It is also likely that the last common ancestor of chordates had common progenitor cell pools for different mesodermal lineages, including the nephrotome. The results we obtained in amphioxus support this notion. Previous studies have suggested that the protocoel of hemichordates and the first somite pair of amphioxus represent homologous structures, as both arise through enterocoely, require fibroblast growth factor as inductive signal, and share common gene expression patterns (*70*). The protocoel gives rise to the nephridia of hemichordates (*71*, *72*). If the protocoel and the first somite pair of amphioxus are homologous, it is possible that Hatschek’s nephridium and the nephridia of hemichordates are also evolutionarily related. This, in turn, would lend further support to the hypothesis that all ultrafiltration-based excretory organs share a single evolutionary origin (*73*).

Our findings further suggest a divergence in the identity of the dorso-lateral dermomyotome and the ventral sclerotome/nephrotome in early vertebrates, as exemplified in the lamprey and in agreement with Della Gaspera *et al.* (*74*). Complete separation of a nephrotome progenitor lineage in a ventro-lateral compartment of the somite likely evolved during early diversification of jawed vertebrates, which agrees with our results in the catshark. The intermediate mesoderm as a distinct tissue and source of the pronephros is thus an evolutionary novelty of osteichthyans, which include bony fish, amphibians, and amniotes. In the latter, the intermediate mesoderm arises from progenitor cells migrating from a specific location within the primitive streak. Grafts of primitive streak fragments from a transgenic green fluorescent protein–expressing mouse into a wild-type mouse revealed an overlap of lateral paraxial mesoderm cells and intermediate mesoderm progenitors (*13*). Furthermore, in chick embryos, several *Hox* genes, but especially *Hoxb4*, which was found to confer the competence of intermediate mesoderm cells to respond to pronephric inductive molecules (*60*), share the same expression pattern in paraxial and intermediate mesoderm, but not in the neural tube or the lateral plate mesoderm (*75*). These results are suggestive of a common developmental identity of early paraxial mesoderm and intermediate mesoderm in amniotes, supporting an evolutionary relationship between these two mesodermal tissues. Integration of these findings highlight an evolutionary trajectory wherein the vertebrate ancestor likely had common somitic progenitors of nephrotome and sclerotome, which later evolved into a distinct nephrotomic compartment of the somite and eventually into a specialized mesodermal tissue. This evolutionary trajectory of the vertebrate mesoderm might be linked to the increasing complexity of vertebrate excretory organs and might have facilitated the control of the regulatory processes associated with the increasing number of morphological transformations required for the subsequent development of the pronephros, the mesonephros, and the metanephros.

## MATERIALS AND METHODS

### Animal procurement and embryo culture

Adult catshark (*S. canicula*) were collected off the coast of Banyuls-sur-Mer (France) and kept at the marine station of Banyuls-sur-Mer (France) at 16°C in oxygenated seawater to retrieve eggs. Embryos were staged according to Ballard *et al.* (*76*). Adult lamprey (*L. fluviatilis*) were spawned at the marine station of Banyuls-sur-Mer (France), and embryos were reared at 16°C as described by Sauka-Spengler *et al.* (*77*) and staged according to tables established for another closely related lamprey species, *Lampetra reissneri*, in Tahara (1988) (*78*). Amphioxus adults (*B. lanceolatum*) were collected in Argelès-sur-Mer (France) and kept at the marine station of Villefranche-sur-Mer (France). Spawning was induced following the protocols described in (*79*), and embryos and larvae were cultured at 19°C according to (*80*) and staged following (*45*). *S. canicula*, *L. fluviatilis*, and *B. lanceolatum* material was fixed and stored according to (*37*).

This work complies with all relevant ethical regulations. For *S. canicula* and *L. fluviatilis*, eggs and embryos were provided by the Aquariology Service of the marine station of Banyuls-sur-Mer (France) (agreement number A6601602). Experimentation on developing catshark and lamprey embryos was carried out at the research unit Biologie Intégrative des Organismes Marins in Banyuls-sur-Mer (France), while experiments on developing amphioxus were conducted at the research unit Laboratoire de Biologie du Développement of the marine station of Villefranche-sur-Mer (France). According to French and European regulations, ethical review and agreement were not required for analyses of catshark, lamprey, and amphioxus specimens, because their study only involved nonfeeding stages of development.

### Pharmacological treatments

Cyclopamine (Sigma-Aldrich, Saint Quentin, France), an inhibitor of Hedgehog signaling activity, was dissolved in dimethyl sulfoxide (DMSO) (Sigma-Aldrich, Saint Quentin, France) to obtain a 10 mM stock solution. The optimal treatment concentration for each model organism was defined by experimental test series with different cyclopamine concentrations. Egg cases of *S. canicula* were injected with a 200 μM cyclopamine solution when the embryo was at stage 12. Embryos were maintained in oxygenated seawater at 16°C until they reached stage 19/20. Embryos of *L. fluviatilis* were treated at stage 12 in culture dishes containing mineral water at a final concentration of 25 μM cyclopamine. The treatment solution was renewed every 3 days, and embryos were kept at 16°C until reaching stage 24. *B. lanceolatum* embryos at the G4 stage (9 hours postfertilization) were treated according to (*81*) in petri dishes containing artificial sea water at a final concentration of 2 μM cyclopamine. Larvae were subsequently reared at 19°C until the L0 stage (42 hours postfertilization). Control treatments for each model were 1:1000 dilutions of DMSO. Following pharmacological treatment, *S. canicula*, *L. fluviatilis*, and *B. lanceolatum* material was fixed and stored according to (*37*).

### Whole-mount chromogenic in situ hybridization

Coding sequences were cloned, and antisense RNA probes were synthesized according to (*77*). The GenBank accession numbers of the genes used for chromogenic in situ hybridization are as follows: *S. canicula Pax2*, EF185884; *S. canicula Lim1*, AY217780; *S. canicula Sox9*, EU241880; *Petromyzon marinus Pax2*, XM032949697; *L. fluviatilis Lim1*, DQ002012; *P. marinus Sox9*, XP_032830402. Whole-mount chromogenic in situ hybridization of *S. canicula*, *L. fluviatilis*, and *B. lanceolatum* embryos were carried out according to protocols from, respectively, Sauka-Spengler *et al.* (*77*), Takio *et al.* (*82*), and Yu and Holland (*83*). Image acquisition was as described in (*37*).

### Histology

Cryo-sections of *S. canicula* and *L. fluviatilis* embryos were carried out either following chromogenic whole-mount in situ hybridization experiments or before immunohistochemistry and in situ HCR analyses. For cryo-sectioning, embryos of *S. canicula* and *L. fluviatilis* were washed in 5% sucrose in phosphate-buffered saline (PBS) for 1 hour before being incubated in 20% sucrose in PBS overnight at 4°C. The next day, embryos were transferred to a solution of 15% sucrose and 7.5% gelatin (Sigma-Aldrich, Saint Quentin, France) and incubated in a 38°C water bath for 1 hour. Embryos were then embedded in this medium on ice, trimmed to the appropriate orientation, and frozen in 2-methylbutane (Sigma-Aldrich, Saint Quentin, France) pretreated with dry ice. The frozen gelatin blocks were mounted on cryostat holders with Optimal Cutting Temperature (OCT) compound (Scigen, Paramount, CA, USA) and sectioned with a Leica CM 1950 cryostat (Leica, Wetzlar, Germany). Sections were placed on microscope slides either for imaging (following whole-mount in situ hybridization) or for further analyses (for subsequent immunohistochemistry or in situ HCR experiments). Before imaging, slides were covered with coverslips and mounted using the VECTASHIELD HardSet mounting medium with DAPI (Vector Laboratories, Newark, CA, USA). Image acquisition was as described in (*37*).

### In situ HCR

Probes for in situ HCR were purchased from Molecular Instruments (Los Angeles, CA, USA), according to gene sequences obtained from National Center for Biotechnology Information using the following accession numbers: *S. canicula Pax1*, EU196402; *S. canicula Pax2*, EF185884; *S. canicula Pax3*, EF185883; *P. marinus Pax2*, XM032949697; *P. marinus LecD*, MT125612; *P. marinus Col2*α*1a*, DQ136024; *B. lanceolatum Pax2/5/8*, MF536418; *B. lanceolatum Pax3/7a*, MF979121; *Branchiostoma floridae Lim1/5*, DQ399521. The sequence for *B. lanceolatum ColA* can be retrieved on the EnsemblMetazoa site (https://metazoa.ensembl.org) using the reference BL01498. The reaction kit, including buffers and hybridization solution, was purchased from Molecular Instruments (Los Angeles, CA, USA), and in situ HCR experiments were conducted according to the Molecular Instruments (Los Angeles, CA, USA) standard protocol. While embryos of *S. canicula* and *L. fluviatilis* were cryo-sectioned before the in situ HCR procedure, as described above, in situ HCR experiments in *B. lanceolatum* were carried out on whole-mount embryos and larvae. Nuclear staining in catshark and lamprey was performed using the VECTASHIELD HardSet mounting medium, which contains DAPI (Vector Laboratories, Newark, CA, USA). Sections of *S. canicula* and *L. fluviatilis* embryos were photographed using a Nikon Eclipse Ti2-E fluorescence microscope (Nikon Europe, Amstelveen, the Netherlands) equipped with an Andor Zyla 4.2 sCMOS camera operated by the NIS-Elements software AR 5.21.03. For amphioxus, nuclear staining was carried out by incubating embryos and larvae in a 1:5000 dilution of Hoechst dye (Thermo Fisher Scientific, Illkirch-Graffenstaden, France) in PBS for 12 hours at 4°C. Following whole-mount in situ HCR, *B. lanceolatum* embryos and larvae were imaged using a Leica SP8 confocal microscope (Leica, Wetzlar, Germany) operated by the Las-X 5.2.2. software. Images were edited using Adobe Illustrator and assembled into final figures.

### Immunohistochemistry

Following cryo-sectioning, slides were washed in 1× PBS and blocked with 5% goat serum and 1% bovine serum albumin in PBS. The primary antibody was diluted in blocking solution and applied to the slides, which were incubated overnight at 4°C. After several washing steps, slides were incubated with secondary antibody in blocking solution for 1 hour at room temperature (RT). Following further washes in 1× PBS, slides were covered with coverslips and mounted with VECTASHIELD HardSet mounting medium with DAPI (Vector Laboratories, Newark, CA, USA). Antibodies used were as follows: anti-Pax2 primary (Abcam ab229318, dilution 1:250) with secondary goat anti-rabbit (Abcam ab150081, dilution 1:250); anti-Pax3 primary (Abcam ab69856, dilution 1:250) with secondary goat anti-mouse (Abcam ab150110, dilution 1:250); anti-Laminin primary (Abcam ab11575, dilution 1:200) with secondary goat anti-rabbit (Abcam ab150081, dilution 1:250); anti-MMP2 primary (Abcam ab86607, dilution 1:200) with secondary goat anti-mouse (Abcam ab150110, dilution 1:250); anti-PCNA primary (Abcam ab29, dilution 1:2000) with secondary goat anti-mouse (Abcam ab150110, dilution 1:250); and anti–N-cadherin primary (Abcam ab18203, dilution 1:1000) (Abcam, Cambridge, UK) with secondary goat anti-rabbit (Thermo Fisher Scientific A-11034, dilution 1:500) (Thermo Fisher Scientific, Illkirch-Graffenstaden, France). Imaging of *S. canicula* and *L. fluviatilis* sections was carried out with a Nikon Eclipse Ti2-E fluorescence microscope (Nikon Europe, Amstelveen, the Netherlands) and an Andor Zyla 4.2 sCMOS camera using the NIS-Elements software AR 5.21.03. Images were edited using Adobe Illustrator and assembled into final figures.

### Whole-mount immunohistochemistry and light sheet imaging of catshark embryos

Catshark embryos were fixed in 4% paraformaldehyde for 24 hours at 4°C, washed with 1× PBS, and gradually transferred to 100% methanol before storage at −20°C until use. For whole-mount immunohistochemistry, embryos were permeabilized through three freeze-thaw cycles consisting of incubation at −80°C for 1 hour followed by 30 min at RT and lastly incubated overnight at 4°C. Embryos were then gradually rehydrated into 1× PBS containing 1% Triton X-100 (PBT) and incubated for 2 hours at RT in blocking solution: PBS containing 1% Triton X-100, 10% fetal calf serum, and 5% DMSO. Primary antibody incubation was carried out in blocking solution for 2 days at RT, followed by overnight washing in PBT. Secondary antibody incubation was performed in blocking solution for an additional 2 days, followed by washing in PBT for 6 hours. The following primary antibodies were used: anti-Pax2 (Biotest PRB-276P-200, dilution 1:400) (Biotest, Dreieich, Germany) and anti-Agrin (DSHB 6D2, dilution 1:10) (DSHB, Iowa City, IA, USA). Secondary antibodies were conjugated with Alexa Fluor 488 and cyanine 3 dye (Cy3) (Jackson 711-545-152 and 715–166-150, respectively, dilution 1:250) (Jackson ImmunoResearch Europe, Ely, UK, references). Stained embryos were embedded in 1.5% agarose prepared in double-distilled water (DDW). Agarose blocks containing the embryos were stored in DDW at 4°C, protected from light. Clearing was performed using RapiClear (RI = 1.52) (SUNJin Lab, Hsinchu City, Taiwan) for 2 hours, repeated twice with solution replacement, followed by gentle shaking overnight at RT. Image acquisition was performed using a light sheet microscope (SmartSPIM, LifeCanvas Technologies, Cambridge, MA, USA). Cleared agarose blocks containing the embryos were mounted on a holder using super glue and placed in a chamber filled with EasyIndex matched immersion oil (LifeCanvas Technologies, Cambridge, MA, USA). Excitation wavelengths were 405, 488, and 561 nm, and emission was detected using 445/58-, 525/50-, and 600/52-nm filters, respectively. Images were acquired using a 3.6× objective [TL4X-SAP, numerical aperture = 0.2, working distance (WD) = 17 mm] (Thorlabs, Puteaux, France), with a spatial resolution of 1.8 μm in the *XY* plane and 2 μm along the *Z* axis, and a Hamamatsu Fusion C14440 sCMOS camera (2048 × 2048 pixels). Images were destriped and stitched, and figures were generated using the Imaris software (Oxford Instruments, Abingdon, UK).
