# Supplementary material for: Unraveling the origin of the vertebrate kidney: Emergence from a somitic compartment
Source: Sci Adv. 2026 Jun 26;12(26):eadr2618. doi: 10.1126/sciadv.adr2618 (PMC13308597; doi:10.1126/sciadv.adr2618)
Supplement: Supplementary file 1 — Figs. S1 to S8 [file sciadv.adr2618_sm.pdf]

Science Advances
AAAS

Supplementary Materials for

Unraveling the origin of the vertebrate kidney: Emergence from a somitic compartment

Pascal Schmidt *et al.*

Corresponding author: Ram Reshef, reshefr@sci.haifa.ac.il

Sci. Adv. **12**, eadr2618 (2026)
DOI: 10.1126/sciadv.adr2618

This PDF file includes:

Figs. S1 to S8

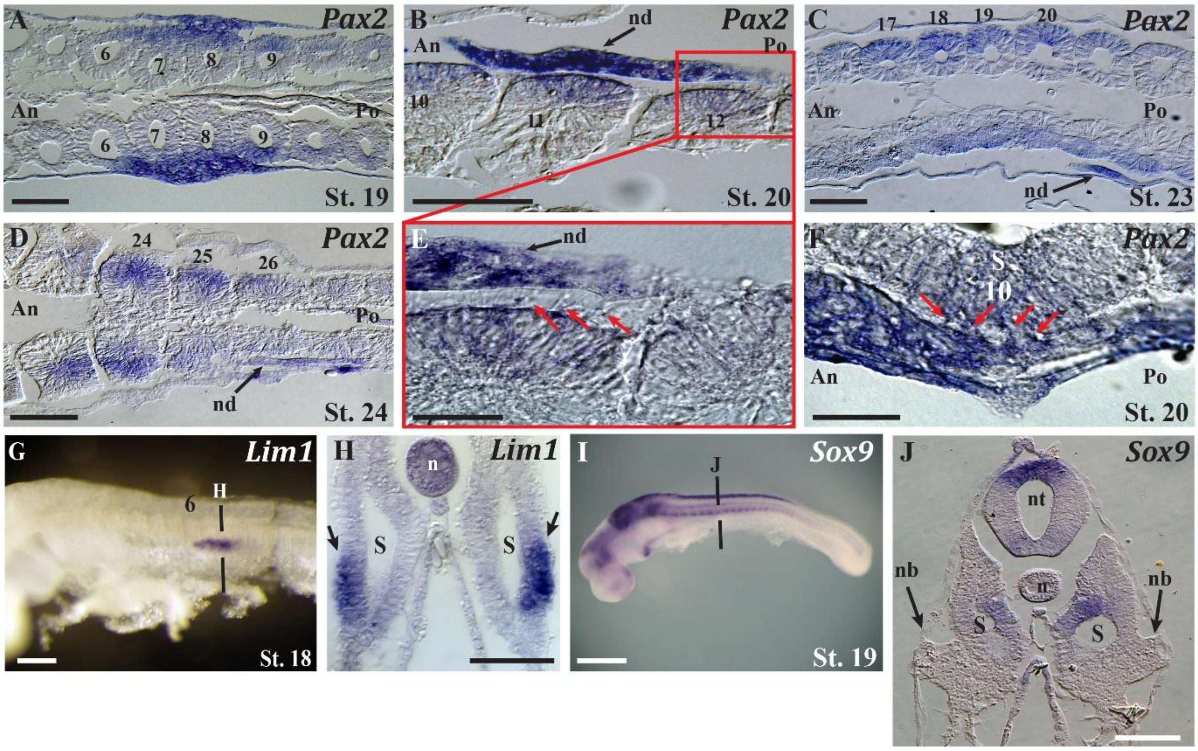

Figure S1. Somitic origin of the catshark pronephros. (A-F) Frontal sections of catshark (*Scyliorhinus canicula*) embryos labeled for *Pax2* mRNA expression by colorimetric *in situ* hybridization at developmental stages 19 **(A)** (n=4), 20 **(B,E,F)** (n=3), 23 **(C)** (n=2), and 24 **(D)** (n=3) at different levels along the anterior-posterior body axis, as indicated by somite numbers. **(G,H)** Expression of *Lim1* mRNA established by colorimetric *in situ* hybridization at developmental stage 18 (n=22), with whole-mount showing segmented expression of the gene **(G)** and with cross-section revealing a ventro-lateral somitic domain highlighted by arrows **(H)**. **(I,J)** Colorimetric *in situ* hybridization for *Sox9* mRNA expression at developmental stage 19 (n=3), showing segmented expression of the gene in whole-mount **(I)** and localization in the sclerotome in cross-section **(J). (A)** is a composite panel of two consecutive frontal sections. Abbreviations: An, anterior; n, notochord; nb, nephric bud; nd, nephric duct; nt, neural tube; Po, posterior; S, somite. Scale bars: **(A-D,G)**: 100 µm; **(E,F)**: 30 µm; **(H,J)**: 50 µm; **(I)**: 400 µm.

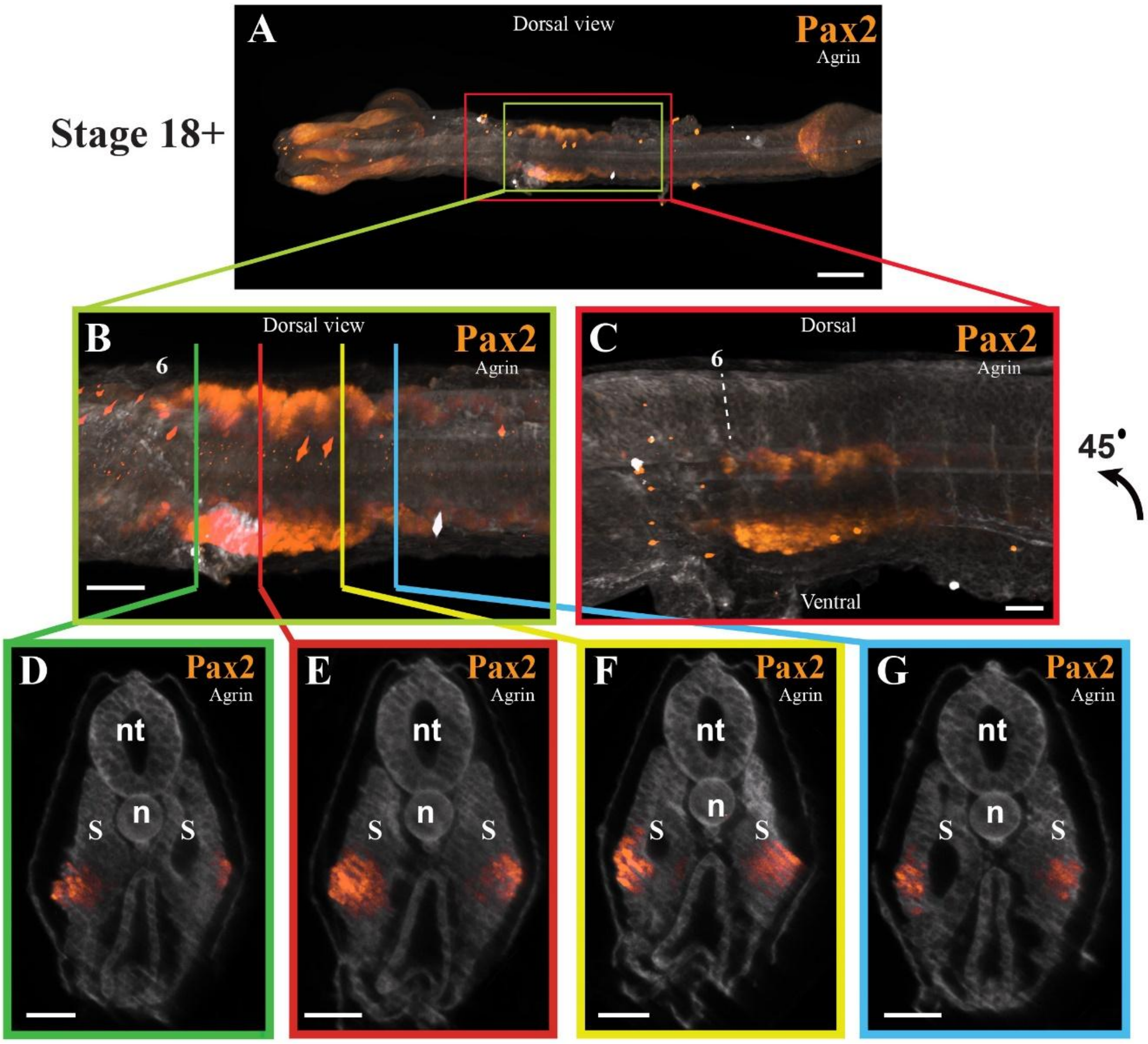

Figure S2. Somitic origin of the catshark pronephros: light sheet microscopy analysis of the stage 18+ catshark embryo. (A) Dorsal view of a stage 18+ catshark (*Scyliorhinus canicula*) embryo following immunohistochemistry for Pax2 (orange) and Agrin (white), a cell membrane marker. Anterior to the left. **(B)** Magnification of the green box in **(A)**. The segmented pattern of the pronephros is shown by Pax2 expression stretching posteriorly from the level of somite 6. **(C)** Magnification of the red box in **(A)** and rotated by 45° to provide a slanted ventral view of the developing pronephros on both the left and right sides of the embryo. **(D-G)** Cross-section images at the levels indicated in **(B)**, which are also highlighted by corresponding colored lines and boxes. Abbreviations: 6, position of somite 6; n, notochord; nt, neural tube; S, somite. Scale bars: **(A)**: 100 µm; **(B-G)**: 50 µm.

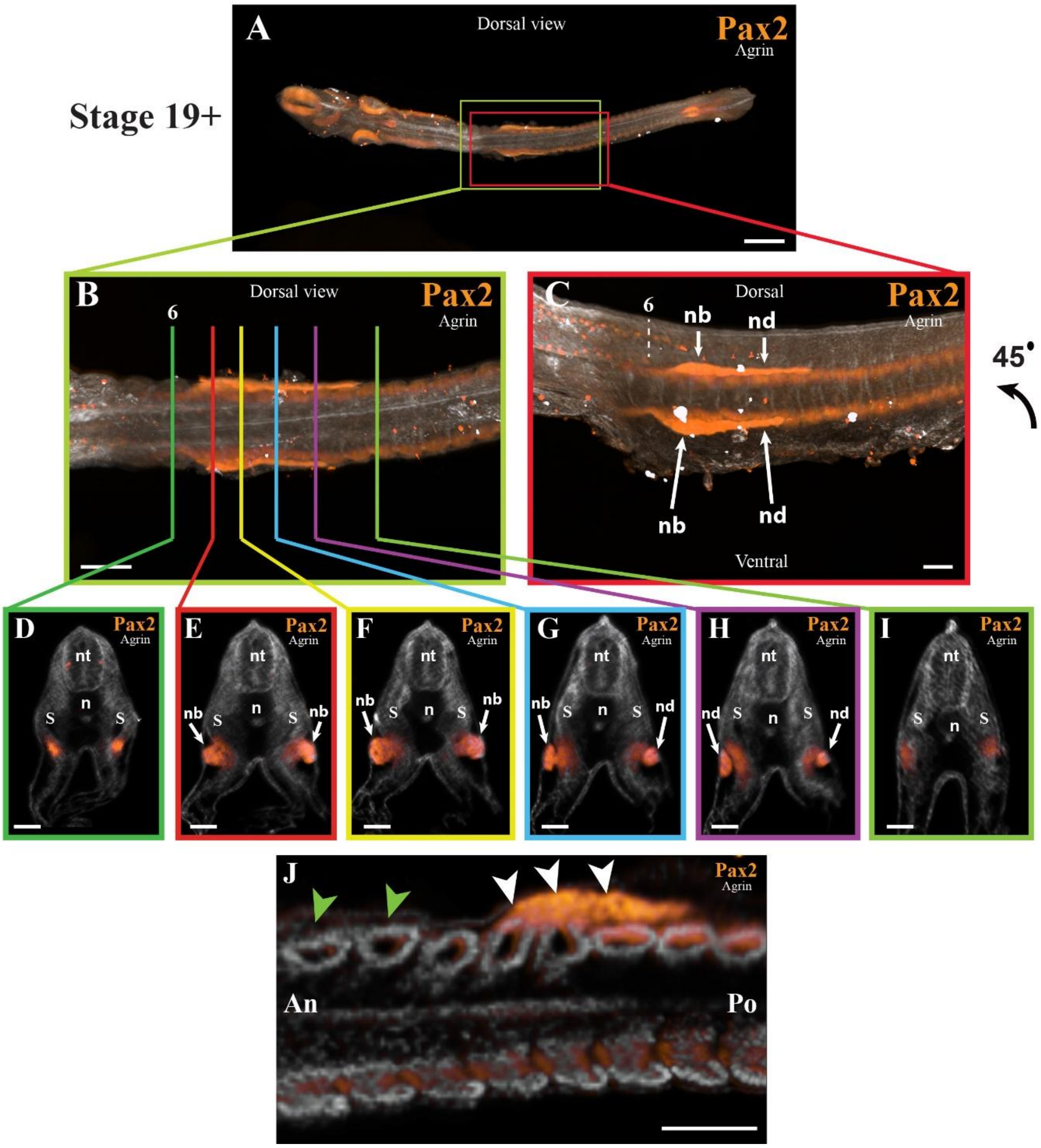

Figure S3. Somitic origin of the catshark pronephros: light sheet microscopy analysis of the stage 19+ catshark embryo. (A) Dorsal view of a stage 19+ catshark (*Scyliorhinus canicula*) embryo following immunohistochemistry for Pax2 (orange) and Agrin (white), a cell membrane marker. Anterior to the left. **(B)** Magnification of the green box in **(A)**. Pronephros budding and posterior extension of the pronephric duct is shown by Pax2 expression stretching posteriorly from the level of somite 6. **(C)** Magnification of the red box in **(A)** and rotated by 45° to provide a slanted ventral view of the developing pronephros on both the left and right sides of the embryo. **(D-I)** Cross-section images at the levels indicated in **(B)**, which are also highlighted by corresponding colored lines and boxes. **(J)** Semi-frontal section showing pronephros budding on the right side. Note the disruption of the lateral wall of the somites at the budding site (white arrowheads), in comparison to somites located more anteriorly in the embryo (green arrowheads). Abbreviations: 6, position of somite 6; An, anterior; n,

notochord; nb, nephric bud; nd, nephric duct; nt, neural tube; Po, posterior; S, somite. Scale bars: **(A,B,J)**: 100 µm; **(C-I)**: 50 µm.

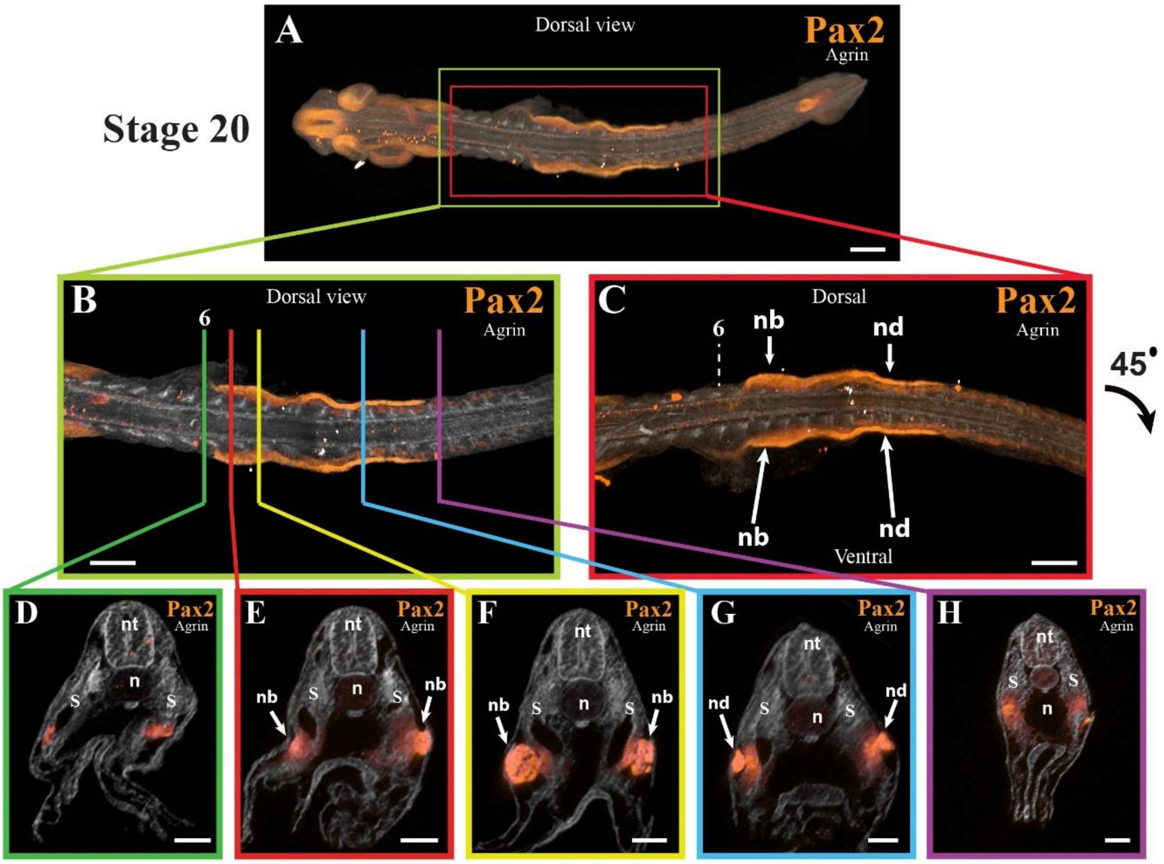

Figure S4. Somitic origin of the catshark pronephros: light sheet microscopy analysis of the stage 20 catshark embryo. (A) Dorsal view of a stage 20 catshark (*Scyliorhinus canicula*) embryo following immunohistochemistry for Pax2 (orange) and Agrin (white), a cell membrane marker. Anterior to the left. **(B)** Magnification of the green box in **(A)**. Pronephros budding and posterior extension of the pronephric duct is shown by Pax2 expression stretching posteriorly from the level of somite 6. **(C)** Magnification of the red box in **(A)** and rotated by 45° to provide a slanted dorsal view of the developing pronephros on both the left and right sides of the embryo. **(D-H)** Cross-section images at the levels indicated in **(B)**, which are also highlighted by corresponding colored lines and boxes. Abbreviations: 6, position of somite 6; n, notochord; nb, nephric bud; nd, nephric duct; nt, neural tube; S, somite. Scale bars: **(A-C)**: 100 µm; **(D-H)**: 50 µm.

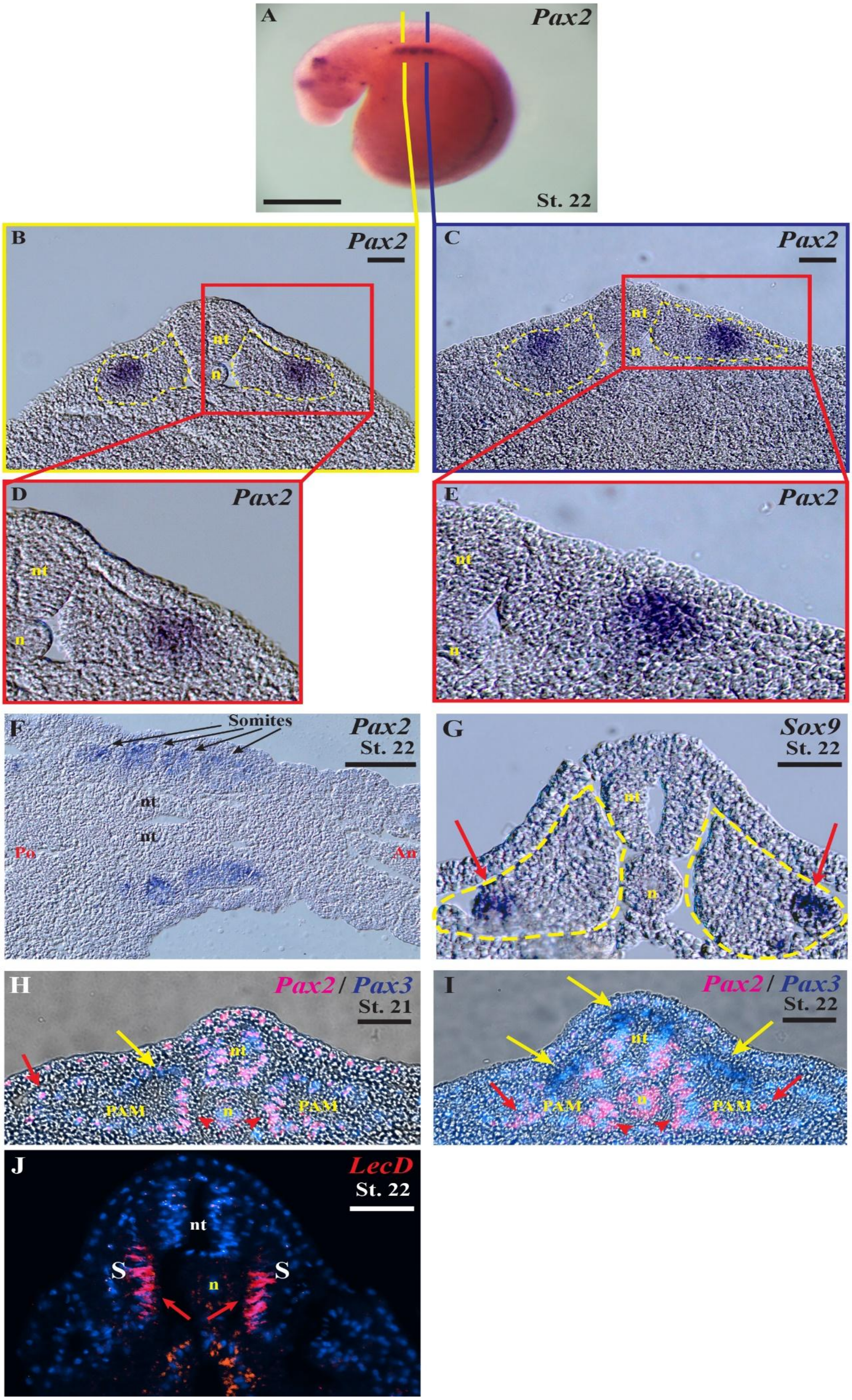
A
Pax2
St. 22
B
Pax2
nt
n
C
Pax2
nt
n
D
Pax2
nt
n
E
Pax2
nt
n
F
Somites
Pax2
St. 22
nt
nt
Po
An
G
Sox9
St. 22
nt
n
H
Pax2 / Pax3
St. 21
nt
PAM
n
PAM
I
Pax2 / Pax3
St. 22
nt
PAM
n
PAM
J
LecD
St. 22
nt
S
n
S

Figure S5. Lamprey pronephros develops from the paraxial mesoderm. (A) Expression of *Pax2* in a whole-mount lamprey (*Lampetra fluviatilis*) embryo at stage 21, assessed by colorimetric *in situ* hybridization (n=43). **(B-E)** Cross-sections of the embryo at the levels indicated in **(A)**, with **(D)** and **(E)** being magnifications of, respectively, **(B)** and **(C)** (n=22). Dotted yellow lines outline the paraxial mesoderm. **(F)** Frontal section of a *Pax2*-labeled embryo at stage 22, with *Pax2* expression in lateral somitic domains revealed by colorimetric *in situ* hybridization (n=12). **(G)** Cross-section of a lamprey embryo at stage 22 showing *Sox9* expression by colorimetric *in situ* hybridization (red arrows) in a lateral domain of the paraxial mesoderm (n=5). The outline of the paraxial mesoderm is marked by a yellow dotted line. **(H,I)** Cross-sections showing results of *in situ* HCR targeting *Pax2* and colorimetric *in situ* hybridization targeting *Pax3/7* at stages 21 (n=3) **(H)** and 22 (n=3) **(I)**. Red arrows highlight *Pax2* expression in the ventro-lateral nephrotome domain and red arrowheads mark *Pax2* expression in the ventro-medial sclerotome domain. Yellow arrows indicate *Pax3/7* expression in the dermomyotome. **(J)** Cross-section showing *LecD* expression in the sclerotome at stage 22 (red arrows), as assessed by *in situ* HCR (n=4). Nuclear label with DAPI in panel **(J)** is shown in blue. Panel A reused from Figure 2B. Abbreviations: An, anterior; n, notochord; nt, neural tube; PAM, paraxial mesoderm; Po, posterior; S, somite; St., stage. Scale bars: **(A)**: 200 µm; **(B,C,G-J)**: 30 µm; **(F)**: 60 µm.

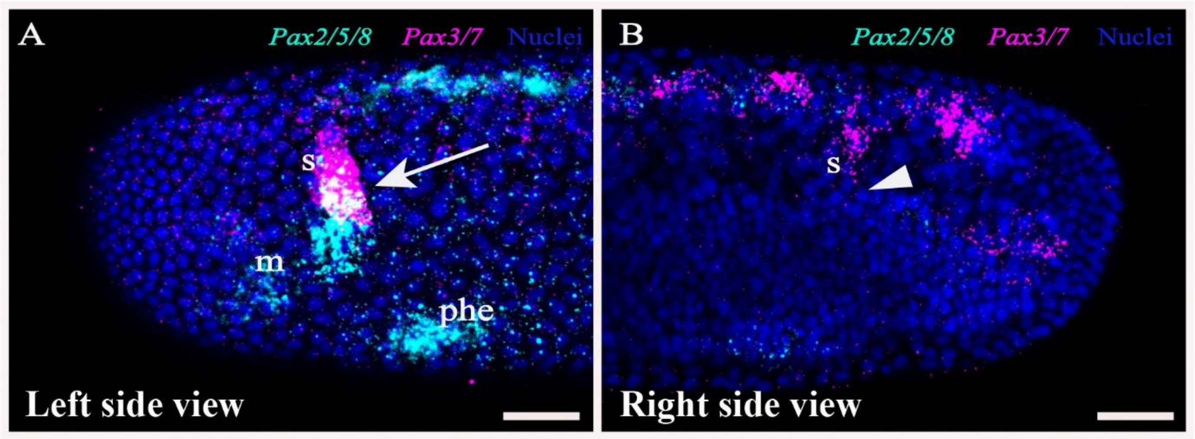

Figure S6. Co-expression of *Pax2/5/8* and *Pax3/7* is limited to the first left amphioxus somite. (A,B) Expression of *Pax2/5/8* and *Pax3/7* of the anterior region of a N5-stage amphioxus embryo of the amphioxus *Branchiostoma lanceolatum*, as revealed by *in situ* HCR. **(A)** Left side view, with anterior to the left, of the anterior region of a N5-stage amphioxus embryo (n=6). Co-expression of *Pax2/5/8* and *Pax3/7* in the ventral compartment of the first left somite is shown in white. Arrow highlights the location of Hatschek's nephridium, co-expressing *Pax2/5/8* and *Pax3/7*. **(B)** Right side view, with anterior to the right, of the anterior region of a N5-stage embryo (n=6). White arrowhead indicates the absence of co-expression of *Pax2/5/8* and *Pax3/7* in the first right somite. Nuclear staining by Hoechst is shown in dark blue. Abbreviations: m, location of the future mouth; phe, pharyngeal epithelium; S, somite. Scale bars: **(A,B)**: 20 µm.

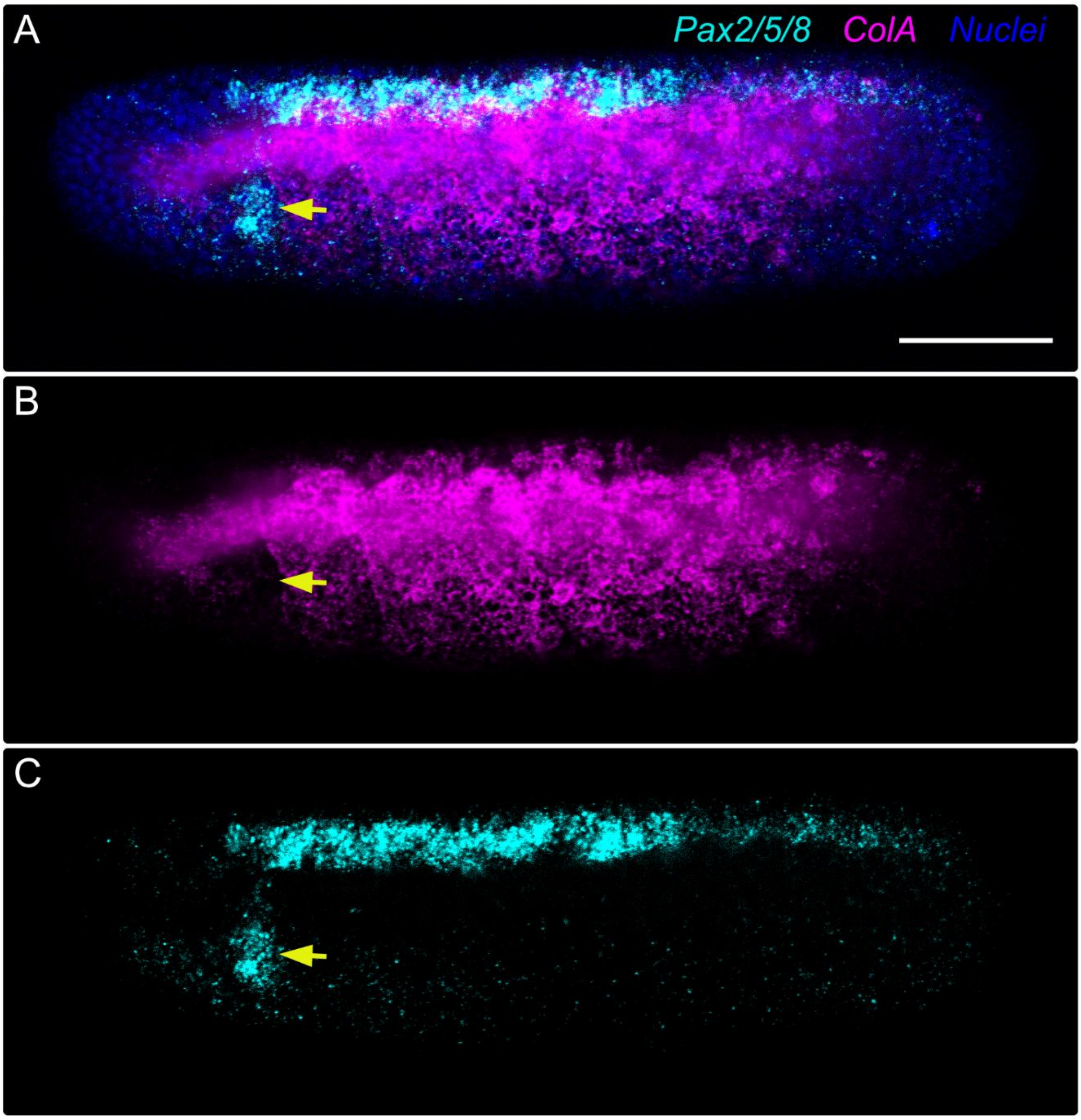

Figure S7. *ColA* is not expressed in the first left somite of amphioxus. (A-C) Gene expression revealed by *in situ* HCR in whole-mount N5-stage embryos of the amphioxus *Branchiostoma lanceolatum*. Embryos with anterior to the left. **(A)** Expression of *Pax2/5/8* and *ColA* on the left side of a N5-stage amphioxus embryo (n=18). **(B)** Expression of *ColA* on the left side of a N5-stage embryo (n=18). **(C)** Expression of *Pax2/5/8* on the left side of a N5-stage embryo (n=18). Arrows highlight the position of Hatschek's nephridium within the first left somite. Nuclear staining by Hoechst in panel **(A)** is shown in dark blue. Scale bars: **(A,B,C)**: 50 µm.

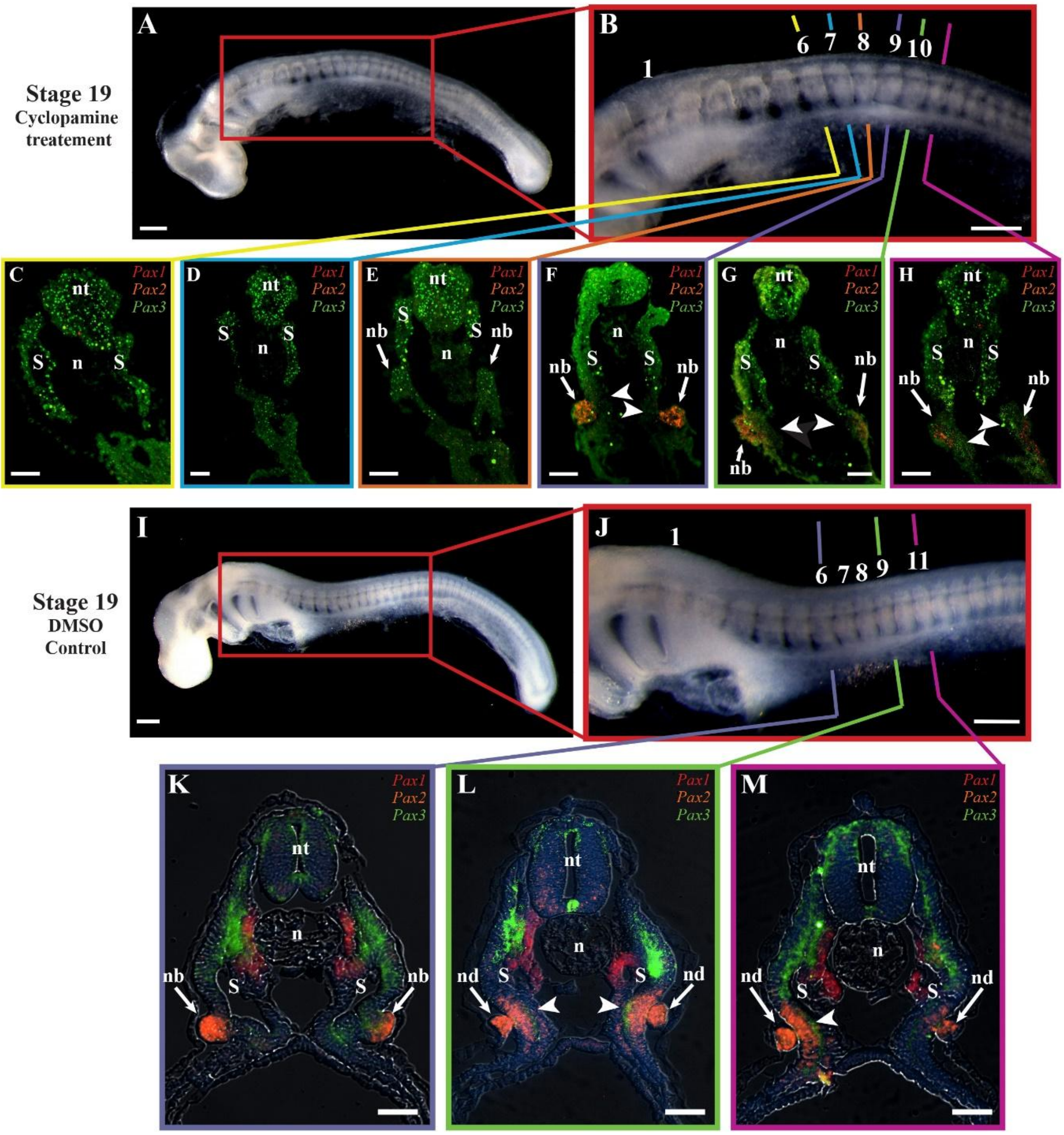

Figure S8. Hedgehog signaling is necessary for the development of the catshark pronephros. Gene expression analysis by *in situ* HCR of catshark (*Scyliorhinus canicula*) embryos following cyclopamine treatment to inhibit Hedgehog signaling. **(A)** Stage 19 catshark embryo following cyclopamine treatment (n=22/32). **(B)** Magnification of the region marked by a red box in **(A)**. Numbers mark somites from anterior to posterior. **(C-H)** *In situ* HCR analysis of *Pax1*, *Pax2*, and *Pax3* expression on cross-sections at the levels indicated in **(B)**. Corresponding colored lines and boxes are indicated, as are somite numbers, where applicable. **(I)** Stage 19 catshark embryo following DMSO control treatment (n=6/7). **(J)** Magnification of the region marked by a red box in **(I)**. Numbers mark somites from anterior to posterior. **(K-M)** *In situ* HCR analysis of *Pax1*, *Pax2*, and *Pax3* expression on cross-sections at the levels indicated in **(J)**. Corresponding colored lines and boxes are indicated, as are somite numbers. Nuclear label with DAPI in panel **(K-M)** is shown in blue. Abbreviations: n, notochord; nb, nephric bud; nd, nephric duct; nt, neural tube; S, somite. Scale bars: **(A,B,I,J)**: 100 µm; **(C-H, K-M)**: 50 µm.
